# Loss of the RNA-binding protein TACO1 causes late-onset mitochondrial dysfunction in mice

**DOI:** 10.1038/ncomms11884

**Published:** 2016-06-20

**Authors:** Tara R. Richman, Henrik Spåhr, Judith A. Ermer, Stefan M. K. Davies, Helena M. Viola, Kristyn A. Bates, John Papadimitriou, Livia C. Hool, Jennifer Rodger, Nils-Göran Larsson, Oliver Rackham, Aleksandra Filipovska

**Affiliations:** 1 Harry Perkins Institute of Medical Research and Centre for Medical Research, University of Western Australia, Nedlands, Western Australia 6009, Australia; 2 Department of Mitochondrial Biology, Max Planck Institute for Biology of Ageing, D-50931 Cologne, Germany; 3 School of Anatomy, Physiology and Human Biology, University of Western Australia, Crawley, Western Australia 6009, Australia; 4 Experimental and Regenerative Neuroscience, School of Animal Biology, University of Western Australia Crawley, Western Australia 6009, Australia; 5 School of Pathology and Laboratory Medicine, University of Western Australia, Crawley, Western Australia 6009, Australia; 6 Victor Chang Cardiac Research Institute, Darlinghurst, New South Wales 2010, Australia; 7 Department of Medical Biochemistry and Biophysics, Karolinska Institutet, Stockholm 17177, Sweden; 8 School of Chemistry and Biochemistry, University of Western Australia, Crawley, Western Australia 6009, Australia

## Abstract

The recognition and translation of mammalian mitochondrial mRNAs are poorly understood. To gain further insights into these processes *in vivo,* we characterized mice with a missense mutation that causes loss of the translational activator of cytochrome oxidase subunit I (TACO1). We report that TACO1 is not required for embryonic survival, although the mutant mice have substantially reduced COXI protein, causing an isolated complex IV deficiency. We show that TACO1 specifically binds the *mt-Co1* mRNA and is required for translation of COXI through its association with the mitochondrial ribosome. We determined the atomic structure of TACO1, revealing three domains in the shape of a hook with a tunnel between domains 1 and 3. Mutations in the positively charged domain 1 reduce RNA binding by TACO1. The *Taco1* mutant mice develop a late-onset visual impairment, motor dysfunction and cardiac hypertrophy and thus provide a useful model for future treatment trials for mitochondrial disease.

## Abstract (web summary)

Mutations in the translational activator of cytochrome c oxidase subunit I (TACO1) causes cytochrome c oxidase deficiency and Leigh Syndrome in patients. Here, the authors characterize mice with a mutation that causes lack of TACO1 expression, identifying a mouse model that could be useful for preclinical trials.

## Graphical abstract

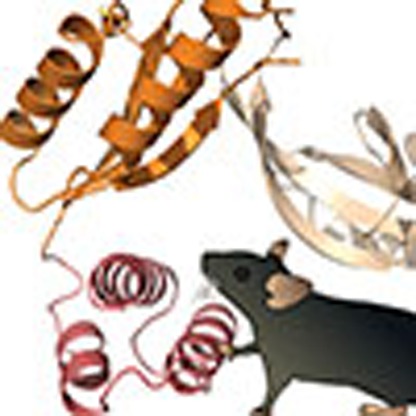

Mitochondria contain their own genome encoding 13 proteins that are part of the membrane-embedded respiratory chain complexes and the ATP synthase required for oxidative phosphorylation (OXPHOS), and 22 tRNAs and 2 rRNAs used by the mitochondrial ribosomes to produce the mitochondrially encoded proteins^1^. In fungi and plants, protein expression in mitochondria is regulated by translational activators that bind to mitochondrial mRNAs, in most cases to the 5′-untranslated regions (UTRs), to facilitate their translation by mitochondrial ribosomes^2^,^3^. In animals, mitochondrial mRNAs lack significant 5′-UTRs^4^ and therefore the translation machinery in mitochondria must have evolved alternate mechanisms to recognize mRNAs and initiate protein synthesis. Although the mechanisms behind the recognition and translation of mitochondrial mRNAs in animals are unclear, recent structures of the small and large mitochondrial ribosomal subunits suggest that the mitochondria-specific ribosomal proteins could play a role in these processes^5^,^6^. Mutations in genes encoding components of the mitochondrial translation machinery are among the most common causes of mitochondrial disease (reviewed in refs 7, 8) but little is known about the mechanisms involved in the regulation of mitochondrial translation.

The translational activator of cytochrome oxidase subunit I (TACO1) was identified when a single-base-pair insertion at position 472 (472insC) was found in patients with late-onset Leigh syndrome (LS) and cytochrome *c* oxidase deficiency^9^. This mutation results in a frame shift that generates a premature stop codon causing loss of the TACO1 protein. TACO1 and its bacterial homologues belong to the domain of unknown function 28 (DUF28) family of proteins that share structural similarity, but their roles in cells are not known^9^.

LS or subacute necrotizing encephalomyelopathy is a neuropathological entity^10^ that can be caused by mutations in the nuclear or mitochondrial genome affecting genes encoding proteins with roles in OXPHOS, respiratory complex assembly, CoQ10 metabolism, mitochondrial gene expression or other aspects of the cellular energy metabolism (reviewed in refs 11, 12). LS patients present with highly variable clinical features affecting many organs that have high-energy demands and are dependent on OXPHOS, including the central nervous system, heart and muscle, leading to neurodegeneration, optic atrophy, ataxia, dystonia and cardiomyopathy^13^. Although LS commonly causes severe symptoms early in life that lead to developmental regression, failure to thrive and often premature death^11^,^13^, patients lacking TACO1 have a late and subtle onset of LS with slowly progressive cognitive dysfunction, dystonia and visual impairment^9^,^14^.

Although TACO1 has been identified as a mitochondrial disease gene, the function of the TACO1 protein is poorly understood. Here we characterize mice that have a homozygous missense point mutation in the *Taco1* gene (*Taco1*^*mut/mut*^), which causes loss of the TACO1 protein. The *Taco1*^*mut/mut*^ mice are born in mendelian proportions and are viable as adults, but have an isolated complex IV deficiency. We show that TACO1 is required for COXI translation, binds the *mt-Co1* mRNA specifically and this interaction is required for association with the mitochondrial ribosome. We also report an atomic structure of TACO1, which shows that the protein consists of three domains in the shape of a hook that form an open state with a tunnel between domains 1 and 3. Guided by the structure, point mutations in the positively charged domain 1 decrease the RNA-binding capacity of TACO1, showing that this domain is interacting with *mt-Co1*. The *Taco1*^*mut/mut*^ mice, similar to human patients, develop a late-onset syndrome with visual impairment, motor dysfunction and cardiac hypertrophy, thus making them a valuable model for the future development of treatments to alleviate symptoms in affected patients.

## Results

### A *Taco1* mutation causes TACO1 loss and complex IV deficiency

**Figure 1:**
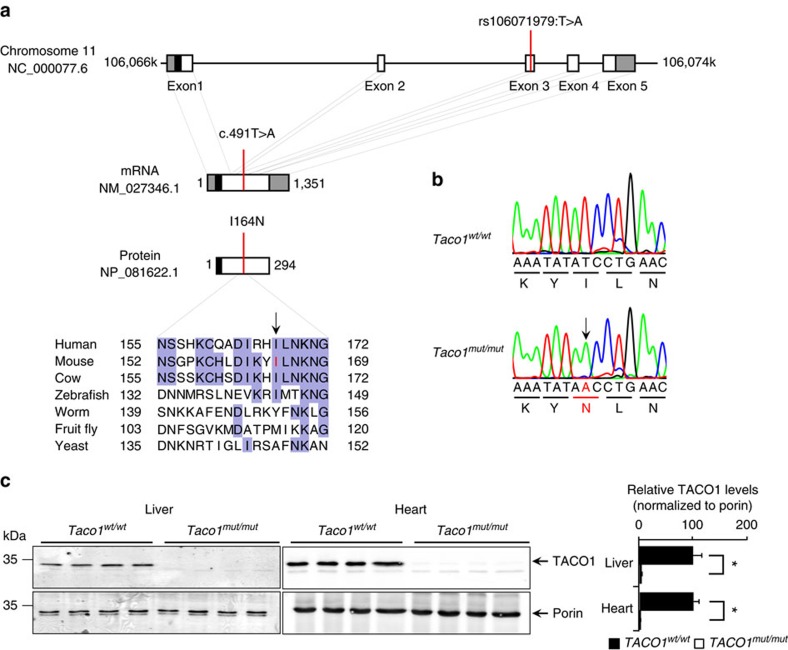
A point mutation in the *Taco1* gene causes destabilization and loss of TACO1 in mice. (**a**) Schematic showing the location of the mutation in the *Taco1* gene, mRNA and protein. The 5′- and 3′-untranslated regions are shown as grey boxes, the predicted mitochondrial targeting sequence is shown as a black box (predicted using MitoProtII^43^). Conservation of the protein sequence surrounding the mutation is highlighted with residues identical to those in the mouse sequence boxed (the mutated isoleucine is shown in red in the mouse sequence). Sequences used were obtained from GenBank at NCBI (human, *Homo sapiens*, NP_057444.2; mouse, *Mus musculus*, NP_081622.1; cow, *Bos taurus*, NP_001192540.1; zebrafish, *Danio rerio*, NP_001076342.1; fruit fly, *Drosophila melanogaster*, NP_609366.2; worm, *Caenorhabditis elegans*, NP_497183.1; yeast, *Saccharomyces cerevisiae*, NP_011535.1) and the alignment was produced using ClustalW2 (ref. 44). (**b**) The mutation was confirmed by Sanger sequencing of PCR amplicons from homozygous *Taco1* mutant mice and matched wild-type littermates. (**c**) TACO1 protein levels were determined in mitochondria isolated from livers and hearts of *Taco1*^*wt/wt*^ and *Taco1*^*mut/mut*^ mice by immunoblotting. Porin was used as a loading control. The data are representative of results obtained from at least 8 mice from each strain.

TACO1 is a ubiquitously expressed protein in a range of different tissues that we have analysed in mice including brain, colon, heart, kidney, liver, thymus, pancreas, skin and testis (Supplementary Fig. 1a). To investigate the *in vivo* role of TACO1, we used a mouse line carrying an ENU-induced T491A point mutation in the *Taco1* gene that converts an isoleucine residue, conserved in vertebrates, at position 164 to asparagine (Fig. 1a) that was identified by whole-exome sequencing^15^. Sanger sequencing shows that the *Taco1*^*mut/mut*^ mice are homozygous for the mutation that is absent in the wild-type (*Taco1*^*wt/wt*^) mice (Fig. 1b). The *Taco1* I164N mutation resulted in a loss of TACO1 protein in the liver and heart mitochondria from *Taco1*^*mut/mut*^ mice indicating that the amino-acid change makes the protein unstable (Fig. 1c).

**Figure 2:**
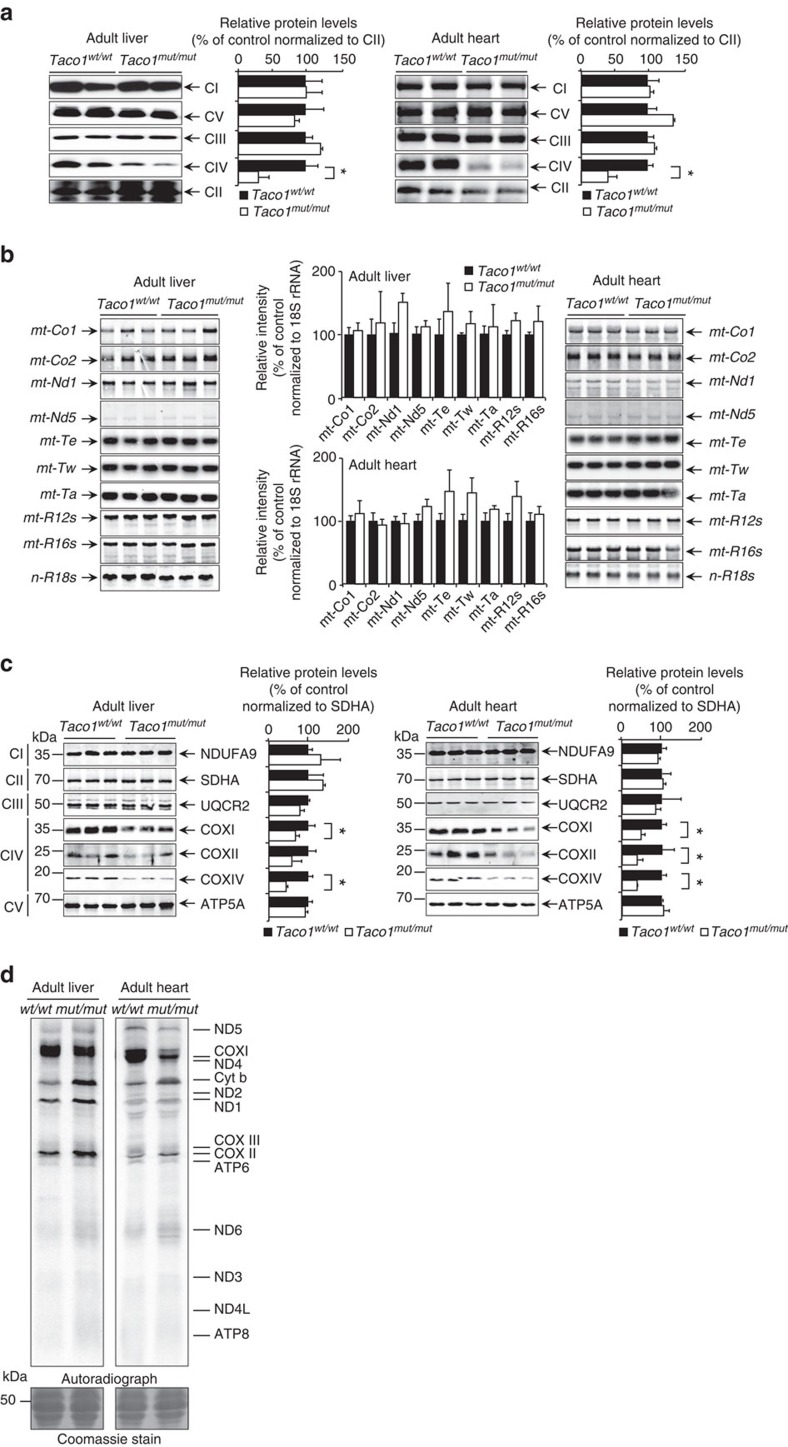
Loss of TACO1 results in isolated complex IV deficiency and specific reduction in COXI translation. (**a**) Mitochondrial proteins (50 μg) isolated from livers and hearts of adult *Taco1*^*mut/mut*^ and *Taco1*^*wt/wt*^ mice were analysed by BN-PAGE and immunoblotting. Specific antibodies representing proteins of each of the mitochondrial complexes were used to compare abundance of complexes in the *Taco1*^*wt/wt*^ and *Taco1*^*mut/mut*^ mice. The data are representative of results obtained from 8 mice from each strain and at least three independent biological experiments. *P<0.05 compared with control treatments by a two-tailed paired Student's *t*-test. (**b**) The *Taco1* mutation does not affect mitochondrial RNA metabolism. The abundance of mature mitochondrial transcripts in mitochondria isolated from adult *Taco1*^*wt/wt*^ and *Taco1*^*mut/mut*^ livers and hearts was analysed by northern blotting. 18S rRNA was used as a loading control. The data are representative of results obtained from at least 8 mice from each strain and three independent biological experiments. *P<0.05 compared with control treatments by a two-tailed paired Student's *t*-test. (**c**) Mitochondrial proteins (20 μg) from adult *Taco1*^*wt/wt*^ and *Taco1*^*mut/mut*^ liver and heart were resolved on SDS–PAGE gels and immunoblotted against antibodies to investigate the steady-state levels of nuclear and mitochondrial-encoded proteins. Porin was used as a loading control. Representative blots are shown of three independent biological experiments. *P<0.05 compared with control treatments by a two-tailed paired Student's *t*-test. (**d**) Protein synthesis in the liver and heart of adult *Taco1*^*wt/wt*^ and *Taco1*^*mut/mut*^ was measured by pulse incorporation of ^35^S-labelled methionine and cysteine. Equal amounts of mitochondrial protein (50 μg) were separated by SDS–PAGE, stained using Coomassie Brilliant Blue to show equal loading and visualized by autoradiography. Representative gels are shown of three independent biological experiments.

We investigated the effects of TACO1 loss on the abundance and integrity of the mitochondrial respiratory complexes in young and adult mice by blue native polyacrylamide gel electrophoresis (BN-PAGE). In mitochondria isolated from livers and hearts of *Taco1*^*mut/mut*^ mice (Supplementary Fig. 1b), we observed a decreased abundance of complex IV, but not of the other respiratory complexes indicating that loss of TACO1 causes an isolated complex IV deficiency in mice. The abundance of the native complexes was determined by immunoblotting following BN-PAGE and confirmed that only complex IV was significantly reduced in both the liver and heart mitochondria from the young and adult *Taco1*^*mut/mut*^ mice compared with the respective control mice (Fig. 2a and Supplementary Fig. 1c).

### TACO1 affects translation of cytochrome *c* oxidase subunit I

We analysed the effects of the *Taco1* mutation on the abundance of mitochondrial RNAs in hearts and livers by northern blotting. There were no significant changes in the levels of mitochondrial mRNAs, tRNAs or rRNAs in the liver and heart mitochondria of young *Taco1*^*mut/mut*^ mice compared with *Taco1*^*wt/wt*^ mice (Supplementary Fig. 2a). Likewise, the steady-state levels of mitochondrial RNAs were not changed in the adult mice revealing that the *Taco1* mutation does not affect their stability and that TACO1 is not required for the stability of the *mt-Co1* mRNA (Fig. 2b).

Next, we analysed the effects of the *Taco1* mutation on the abundance of mitochondrial proteins by immunoblotting in both young and adult mice. The levels of the mitochondrially encoded COXI were decreased in the young *Taco1*^*mut/mut*^ mice in both the heart and the liver (Supplementary Fig. 2b), and in the adult *Taco1*^*mut/mut*^ mice, COXII was reduced in addition to COXI (Fig. 2c). The nuclear encoded COXIV was reduced in both young and adult *Taco1*^*mut/mut*^ mice compared with the *Taco1*^*wt/wt*^ mice, likely in a retrograde response to the sustained reduction of the mitochondrial cytochrome *c* oxidase polypeptides (Fig. 2c and Supplementary Fig. 2b). The abundance of protein subunits from the other complexes remained unchanged (Fig. 2c and Supplementary Fig. 2b), consistent with the specific complex IV decrease as a result of the *Taco1* mutation. This indicates that the *Taco1* mutation affects the production and consequently the abundance of cytochrome *c* oxidase in mice.

Analysis of mitochondrial translation using ^35^S-methionine and cysteine incorporation into newly synthesized proteins revealed specific reduction in the synthesis of COXI in mitochondria of young (Supplementary Fig. 2c) and adult (Fig. 2d) *Taco1*^*mut/mut*^ mice. The reduction of COXI in the mutant mice corroborates previous findings in human cells carrying the *TACO1* mutation that TACO1 is important for the specific translation of the *MT-CO1* mRNA^9^. We observed changes in both mitochondrial DNA (mtDNA) and nuclear encoded polypeptides of cytochrome *c* oxidase, but not in polypeptides of the other respiratory complexes, indicating that only the biogenesis of complex IV is compromised (Fig. 2a and Supplementary Fig. 2c). Cytochrome *c* oxidase is comprised of distinct subcomplexes, which form in a stepwise manner; insertion of COXI in the inner membrane is required for the initial formation of subcomplex 1 (S1) followed by the formation of subcomplex 2 (S2), comprised of COXI and COXIV, thereafter the S3 and S4 subcomplexes are formed to complete the assembly of the entire cytochrome *c* oxidase complex^16^. The reduced translation of COXI in the *Taco1*^*mut/mut*^ mice is rate limiting for the initial stage of complex IV assembly resulting in decreased levels of this complex.

### TACO1 binds *mt-Co1* mRNA and associates with the ribosome

**Figure 3:**
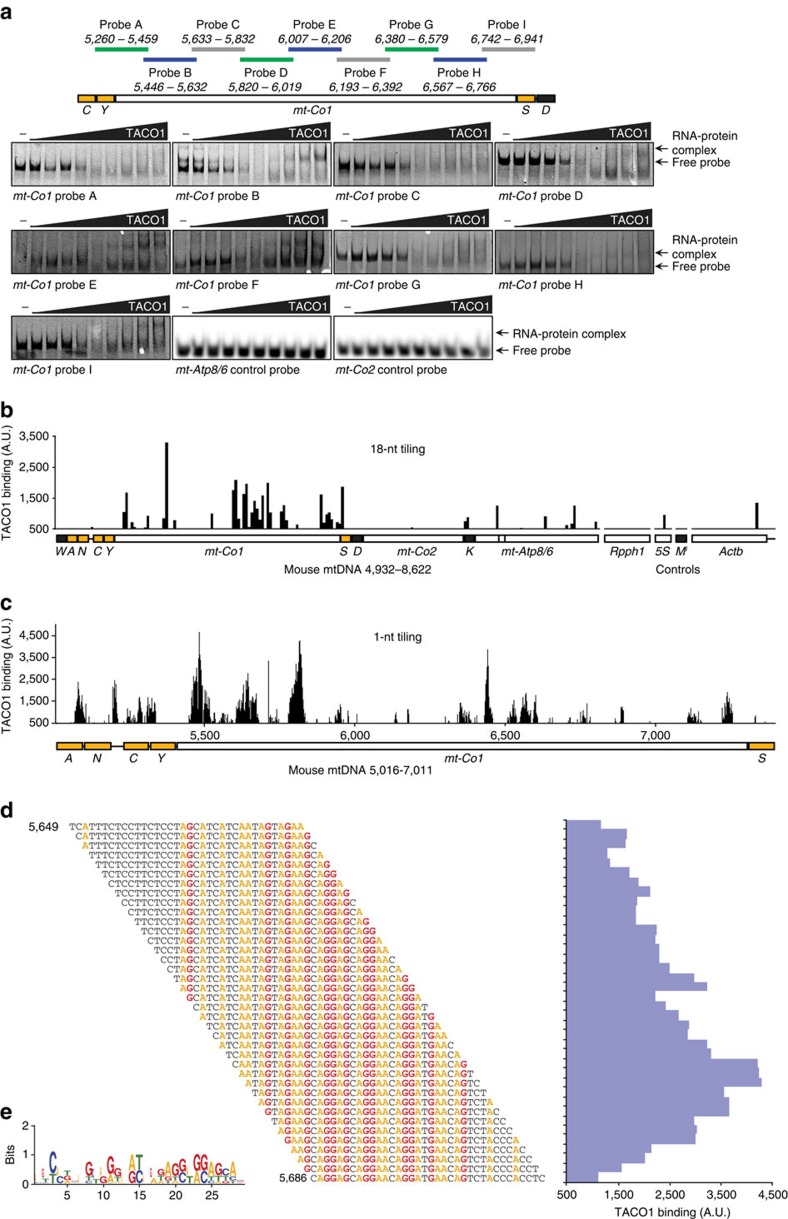
TACO1 is a *mt-Co1* mRNA-binding protein. (**a**) A set of partially overlapping RNA probes was used in RNA electrophoretic mobility shift assays (RNA EMSA). The locations of RNA probes within the *mt-Co1* gene region in the mouse mtDNA are shown schematically. RNA EMSA analyses were performed with increasing concentrations of purified TACO1 protein. (**b**) Identification of TACO1-binding sites within the *mt-Co1* region of mouse mtDNA using a custom RNA tiling microarray. The mtDNA region between nt 4,932–8,622 was tiled in 18 nt increments. The nuclear RNase P RNA (*Rpph1*), 5S rRNA (*5S*), a nuclear encoded initiator tRNA-Met (*M*^*i*^) and 500 nt of the 3′ end of the β-actin mRNA (*Actb*)-coding region and the RNA localization signal within its 3′-UTR were used as controls. (**c**) Single-nucleotide RNA tiling microarray analysis of the TACO1-binding sites within *mt-Co1*. (**d**) Part of the microarray showing binding of TACO1 to sequences sampled at 1-nt intervals through a TACO1-binding site within *mt-Co1* (nt 5,649–5,721). The aligned sequences of the RNA probes for which TACO1 has the greatest affinity are shown to contain guanine (G) and adenine (A) residues, coloured in red and yellow, respectively. (**e**) The consensus binding site for TACO1 within *mt-Co1* was identified using multiple EM for motif elicitation (MEME) analysis and is shown as a sequence logo^45^. The 79 probes with signal above 2,000 A.U. from the single-nucleotide RNA tiling microarray were used for the analysis and the discovered motif occurred with *P*=1.6e-184.

Because we observed that TACO1 specifically affects the translation of the *mt-Co1* mRNA (Fig. 2d), we investigated if TACO1 is an RNA-binding protein that can bind the *mt-Co1* mRNA. We investigated the association of recombinant mouse TACO1 protein using RNA fragments spanning the entire length of the *mt-Co1* mRNA by RNA electrophoretic mobility shift assay (EMSA) (Fig. 3a). We found that TACO1 binds the mRNA at multiple distinct regions relative to *mt-Atp8* and *mt-Co2* that were used as controls suggesting that TACO1 is an RNA-binding protein, which is required for the efficient translation, but not stability of *mt-Co1* mRNA. We identified that TACO1 binds most strongly to probes that correspond to specific regions of *mt-Co1* mRNA between nucleotides 5,446–5,632 and 6,007–6,392. Next, we used an RNA tiling array to investigate the specific binding sites of TACO1. The array was designed to coarsely scan a large area of mtRNA and control cytoplasmic and nuclear RNAs in 36-base successive RNAs with an 18-bp shift per measurement (Fig. 3b). Single-base-pair shifts were then used for a high-resolution scan of the *mt-Co1* mRNA (Fig. 3c). TACO1 bound the array in only a few places and binding was sustained reproducibly with the movement of the experimental window in the high-resolution scan. Interestingly, TACO1 bound at multiple sites in the *mt-Co1* mRNA confirming the RNA EMSA results. This indicates that a productive and functional association might result from cumulative binding at multiple sites enabling *mt-Co1* mRNA to be controlled despite the presence of a few low-affinity-binding sites elsewhere in the mitochondrial transcriptome. The single-nucleotide tiling array of *mt-Co1* identified potential minimal binding sites of TACO1 within this mRNA that are predominantly concentrated at the 5′ end (Fig. 3c,d). Also these correlate well with the binding regions identified within the *mt-Co1* by the RNA EMSAs. We analysed the binding specificity of TACO1 using the multiple Em for motif elicitation and identified that TACO1 has an affinity for adenine-guanine-rich sequences (Fig. 3e) that are enriched in the *mt-Co1* mRNA compared with other mitochondrial mRNAs. Next, we carried out immunoprecipitation of FLAG-tagged TACO1 or GFP control from mouse NIH-3T3 cells in the presence or absence of the dithiobis[succinimidyl propionate] crosslinker and investigated their association with the *mt-Co1* mRNA (Supplementary Fig. 3a). We found that TACO1 associates transiently with the *mt-Co1* mRNA because this association was not found in cells that were not crosslinked compared with control. In the presence of crosslinks, we also detected 12S rRNA and to a lesser degree 16S rRNA, but not *mt-Atp8/6* mRNA (Supplementary Fig. 3a).

**Figure 4:**
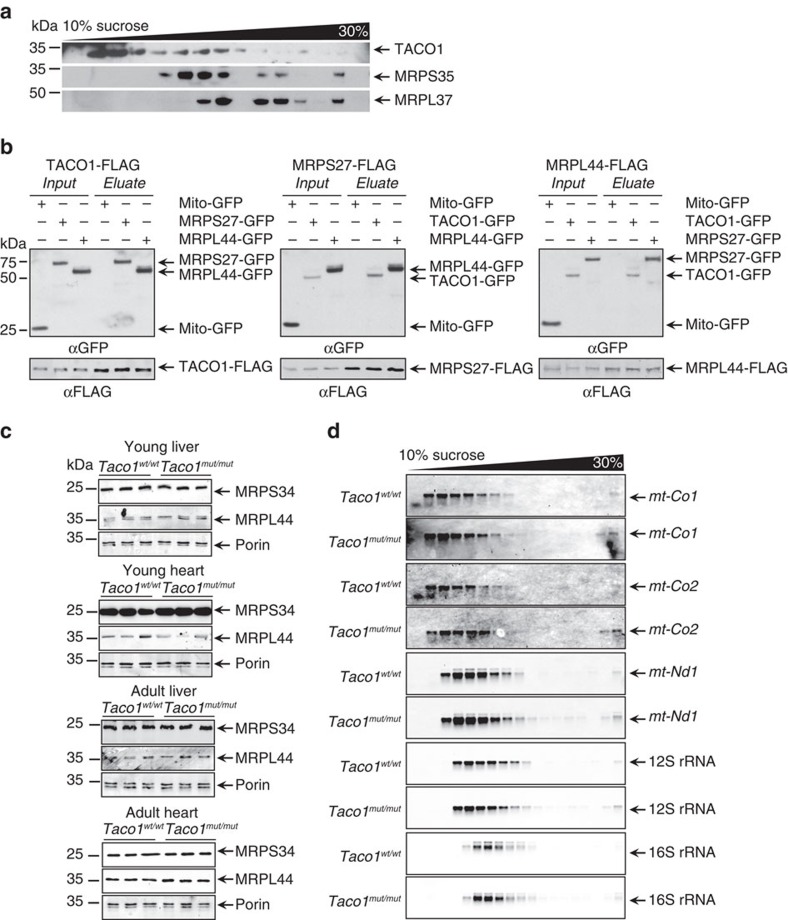
Low levels of TACO1 associate with the mitoribosome. (**a**) A continuous sucrose gradient was used to determine TACO1 distribution relative to mitochondrial ribosomal protein markers of the small (MRPS35) and large (MRPL37) ribosomal subunits by immunoblotting with specific antibodies. (**b**) Immunoprecipitation of FLAG-tagged TACO1, MRPS27 and MRPL44 from NIH-3T3 cells expressing either mitochondria targeted GFP, or GFP-tagged MRPS27, MRPL44 or TACO1, followed by immunoblotting with an anti-GFP antibody and an anti-FLAG antibody to detect the immunoprecipitated proteins. (**c**) Mitochondrial ribosomal protein abundance was measured by immunoblotting in mitochondria isolated from hearts and livers of young and old *Taco1*^*mut/mut*^ and *Taco1*^*wt/wt*^ mice. (**d**) The distributions of mitochondrial mRNAs, 12S and 16S rRNAs in sucrose gradients were analysed by northern blotting. The data are typical of results from at least three independent biological experiments.

As mitochondrial mRNAs lack 5′-UTRs that enable the recruitment of the ribosomal subunits to initiate translation, we investigated if TACO1 associates with the mitochondrial ribosome to facilitate the translation of COXI. Resolution of mitochondrial ribosomes using sucrose gradients revealed that low levels of TACO1 predominantly co-migrate with the small subunit (Fig. 4a), but low levels are also found with the monosome suggesting that TACO1 may facilitate the initiation of translation of *mt-Co1*. To corroborate this finding, we carried out immunoprecipitation of FLAG-tagged TACO1 expressed in NIH-3T3 cells following crosslinking, to show that TACO1 associates with the mitochondrial ribosome (Fig. 4b). A reciprocal immunoprecipitation of either FLAG-tagged MRPS27 or FLAG-tagged MRPL44 confirmed their association with TACO1 (Fig. 4b). We used the association between the small and large ribosomal proteins as a positive control for their interaction. Furthermore, mass spectrometry analyses of endogenous proteins associated with TACO1-FLAG, compared with GFP-FLAG, revealed an enrichment of proteins directly involved in mitochondrial translation as well as mitochondrial ribosomal proteins that were also detected by immunoblotting (Supplementary Fig. 3b,c).

Because of its association with the mitochondrial ribosome, we investigated the effects of the *Taco1* mutation on the abundance of mitochondrial ribosomal proteins from the large and small subunit in liver and heart mitochondria from young and adult *Taco1*^*mut/mut*^ and *Taco1*^*wt/wt*^ mice (Fig. 4c and Supplementary Fig. 3d). There was no change in the abundance of MRPS34 and MRPL44 that were used as marker proteins of the small and large ribosomal subunits, respectively, indicating that TACO1 is not required for the steady-state levels of mitoribosomes. Next, we analysed if the *Taco1* mutation affects the association of mitochondrial transcripts within mitoribosomes using sucrose gradients followed by northern blotting (Fig. 4d). We found that the distribution and abundance of mitochondrial mRNAs and rRNAs are comparable in the *Taco1*^*mut/mut*^ and *Taco1*^*wt/wt*^ mice indicating that this protein is not required for the recruitment of *mt-Co1* mRNA to the mitoribosome. We conclude that the transient binding of TACO1 to the *mt-Co1* mRNA is necessary to stimulate the efficient translation of the COXI polypeptide.

### Structure of TACO1

**Figure 5:**
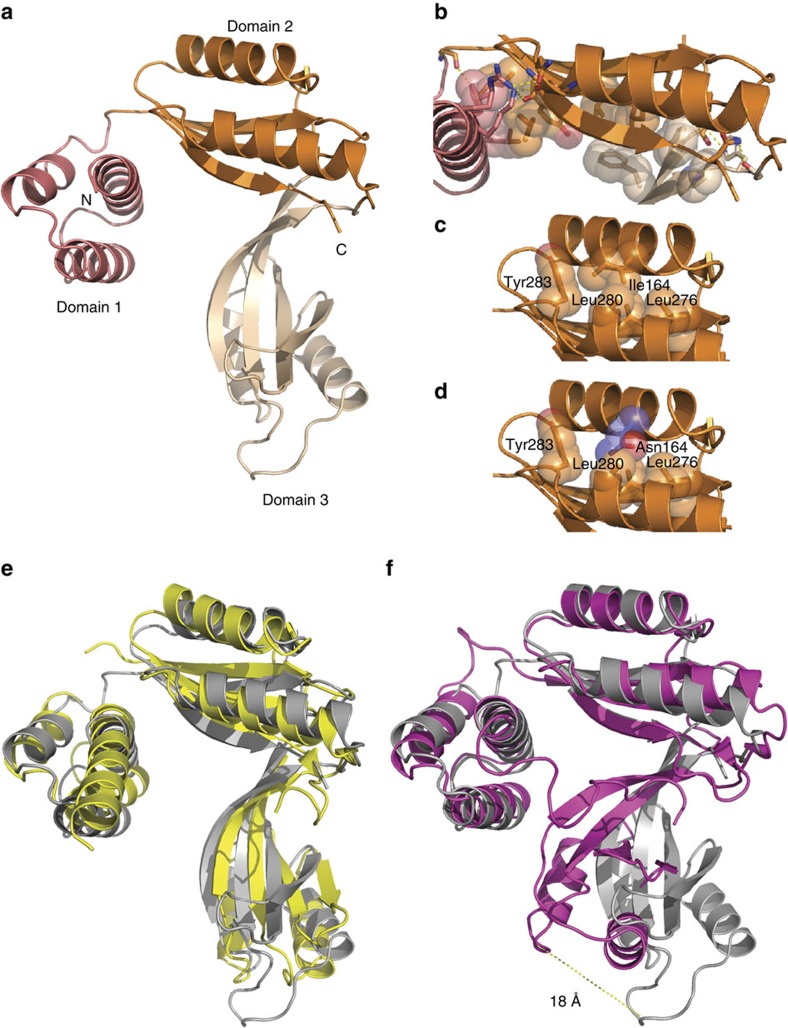
Structure of TACO1 and similarity to bacterial DUF28 family members. (**a**) Front view of TACO1 is in cartoon representation with domain 1 coloured in salmon, domain 2 in orange and domain 3 in wheat, respectively. The N- and C-terminals are indicated. (**b**) Interacting polar residues at the interface between domains are shown as sticks and hydrophobic residues as spheres. (**c**) Intra domain 2 interactions. Leu 164 and surrounding hydrophobic residues are shown as spheres. (**d**) The Asn 164 mutant amino acid and surrounding hydrophobic residues are shown as spheres. (**e**) Superimposition of TACO1 (grey) with Cbu1566 from *Coxiella burnetii* (yellow, PDB ID code 4F3Q)^18^. (**f**) Superimposition of TACO1 (grey) with Aq1575 from *Aquifex aeolicus* (magenta, PDB ID code 1LFP)^19^. The structural alignment was performed using the secondary-structure matching (SSM) method.

To gain molecular insight into TACO1 and its RNA-binding mode, we crystallized the mature form of mouse TACO1 and determined its structure to 2.0 Å resolution by selenomethionine SAD (Supplementary Table 1). The structure (residues 74–294) has the shape of a hook that is comprised of three domains (Fig. 5a). Residues 27–73 were disordered, probably due to pronounced flexibility. Domain 1 is composed of three α-helices forming a helical bundle and domains 2 and 3 each contain four β-strands that face two α-helices forming 2-layer (αβ)-sandwiches (Fig. 5a and Supplementary Fig. 4). The centrally located domain 2 provides a platform for contacts with domains 1 and 3. Both interfaces are built up by hydrophobic interactions flanked by polar residues that additionally contribute to keep the structure intact by forming hydrogen bonds (Fig. 5b and Supplementary Fig. 5). In contrast, no interactions are formed between domains 1 and 3. Instead, a large tunnel with approximate dimensions 23 × 14 × 12 Å^3^ is formed between them. Hydrophobic residues within each domain provide a hydrophobic core crucial for their stability (Fig. 5c). Even a single amino-acid substitution disrupting the hydrophobic core could be sufficient to cause the protein to collapse. This is obviously the consequence of the I164N mutation in the mutant mouse, which resulted in loss of TACO1 (Fig. 1c). In the mutant protein, the side chain of the asparagine residue would face a hydrophobic patch between the two alpha helices of domain 2, causing repulsion between them (Fig. 5d).

### Structural comparison of TACO1 to bacterial DUF28 proteins

A search for structural homologues to TACO1 using Dali^17^ revealed significant matches to YebC and other bacterial proteins belonging to the DUF28 family (Supplementary Table 2). Four proteins contained all three domains present in TACO1, but their functions remain unknown. We superimposed the structure of TACO1 with the two best matches in the DALI search, Cbu1566 from *Coxiella burnetii* (Fig. 5e)^18^ and Aq1575 from *Aquifex aeolicus* (Fig. 5f)^19^. The root-mean-square deviation (RMSD) for the structural superimpositions are 3.1 Å for 234 residues in Cbu1566 and 5.1 Å for 243 residues in Aq1575 (Supplementary Table 2). Both bacterial proteins superpose well with TACO1 in domain 2 with more pronounced differences in the other domains. Compared with TACO1, there is a large extension of the first α-helix at the N-terminus in Cbu1566 from 5 to 9 turns and domain 3 in Aq1575 is shifted ∼18 Å towards domain 1. This large shift enables contacts to be formed between domains 1 and 3 in Aq1575 and shows that proteins that belong to the DUF28 family can exist in an open state with a tunnel (TACO1) and a closed state (Aq1575). TACO1, with its open state, appears more flexible with a rather high averaged B-factor of ≈57 Å^2^ (Supplementary Table 1) in comparison to ≈35 Å^2^ for Aq1575, suggesting that the interaction between domains 1 and 3 provides rigidity to the structure.

### TACO1 binds RNA via its positively charged domain 1

**Figure 6:**
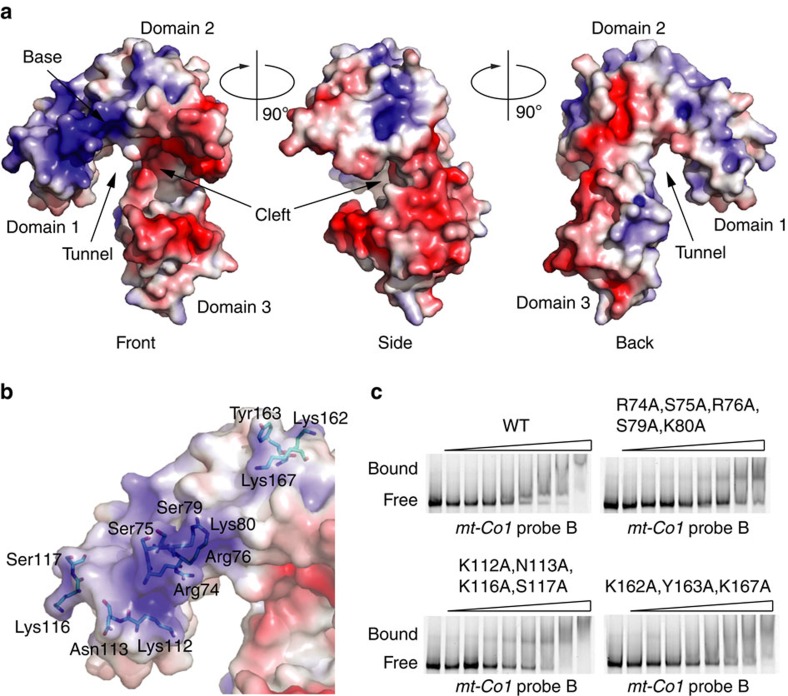
Surface charge distribution and RNA binding of TACO1. (**a**) Front, side and back views of the molecular surface are coloured by the local electrostatic potential (blue, +5 kT; red −5 kT). (**b**) Front view of the surface of TACO1 with mutated residues shown as sticks. (**c**) RNA EMSA of TACO1 mutants. Analyses were performed with increasing concentrations of purified TACO1 protein. Free and bound RNAs are indicated.

TACO1 has a highly asymmetrical surface charge distribution with a gradual change in charge from domains 1 to 3 (Fig. 6a). Domain 1 is widely positively charged, domain 2 has both positively and negatively charged patches and domain 3 is predominantly negatively charged. In addition to the tunnel between domains 1 and 3 there is a cleft located between domains 2 and 3 with approximate dimensions 20 × 10 × 10 Å^3^. The residues surrounding the cleft are not likely to participate in RNA binding, as they are predominantly acidic (Fig. 6a, Front, Side). In contrast, on the domain 1 side of the tunnel, there is a highly positively charged patch at the base of the hook formed by the first N-terminal residues that could be seen in the structure along with additional residues spread throughout the domain (Fig. 6a, Front, Back). We hypothesized that the positively charged surface along domain 1 that continues further along domain 2 is likely an RNA-binding region. To test this hypothesis, we introduced point mutations along the predicted RNA-binding region (Fig. 6b) and performed RNA EMSAs (Fig. 6c). Indeed, RNA binding was severely compromised with a quintuple mutation (R74A, S75A, R76A, S79A and K80A) at the base of the hook of domain 1. In contrast, RNA binding was affected to a lesser extent with a quadruple mutation (K112A, N113A, K116A and S117A) in domain 1 and a triple mutation (K162A, Y163A and K167A) in domain 2, indicating that the base of domain 1 contacts RNA.

### The *Taco1* mutation lowers cytochrome *c* oxidase activity

**Figure 7:**
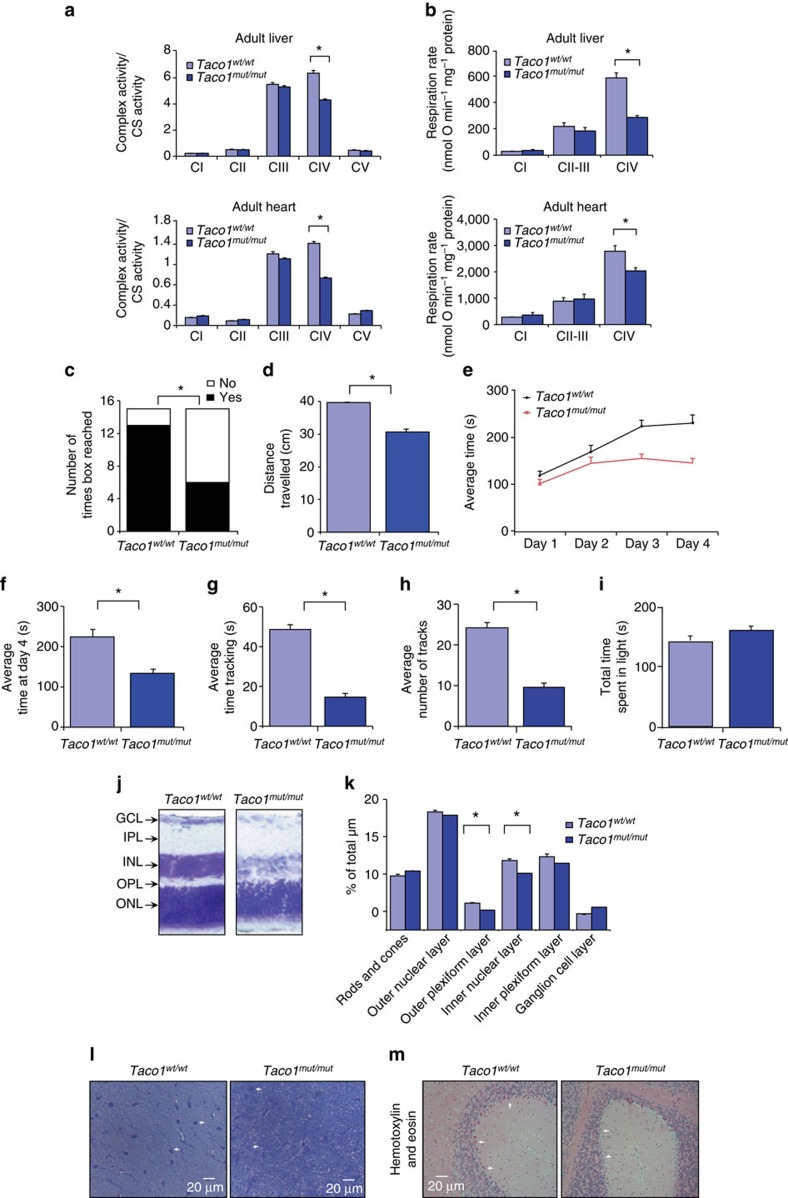
*Taco1*^*mut/mut*^ mice have reduced complex IV enzyme activity, motor dysfunction and visual impairment. (**a**) Respiratory complex activities were normalized to citrate synthase activity in mitochondria from *Taco1*^*wt/wt*^ (*n*=8) and *Taco1*^*mut/mut*^ mice (*n*=8). Data are means±s.e.m. of three to four separate experiments; *P<0.05 compared with control treatments by a two-tailed paired Student's *t*-test. (**b**) Reduced state 3 respiration in *Taco1*^*mut/mut*^ mice compared with *Taco1*^*wt/wt*^ and *Taco1*^*mut/mut*^ was measured using an OROBOROS oxygen electrode. Data are means±s.e.m. of three to four separate experiments; *P<0.05 compared with control treatments by a two-tailed paired Student's *t*-test. Quantification of behavioural studies evaluating number of times the box was reached in hanging wire experiments (**c**) and distance travelled along the wire in hanging wire experiments (**d**) comparing *Taco1*^*wt/wt*^ (*n*=5) and *Taco1*^*mut/mut*^ (*n*=5) mice. (**e**) Rotarod results measured in seconds spent on the rotarod over 4 days to show improvement and learning ability. (**f**) Time spent on the rotarod over 4 days to analyse motor function and learning ability. (**g**) Comparison of *Taco1*^*wt/wt*^ (*n*=5) and *Taco1*^*mut/mut*^ (*n*=5) tracking ability using optokinetic drum, measured in number of times spent tracking. (**h**) Comparison of *Taco1*^*wt/wt*^ and *Taco1*^*mut/mut*^ tracking ability measured in number of tracks performed. (**i**) Comparison of time spent in light versus dark in *Taco1*^*wt/wt*^ (*n*=5) and *Taco1*^*mut/mut*^ (*n*=5) mice. All behavioural data are means±s.e.m.; *P<0.01 compared with controls by a two-tailed Student's *t*-test. (**j**) Cresyl violet staining of 20 μm eye slices of adult *Taco1*^*mut/mut*^ mice (*n*=5) compared with *Taco1*^*wt/wt*^ mice (*n*=5) at × 40 magnification. (**k**) Quantitative analyses of the layers of the cresyl violet-stained slides were determined as a percentage of total μm using Image J. (**l**) Cresyl violet/toluidine blue staining of optic nerves from *Taco1*^*wt/wt*^ (*n*=5) and *Taco1*^*mut/mut*^ (*n*=5) mice visualized at × 100 magnification. (**m**) Scattered foci of mild cell loss and shrinkage of Purkinje cells are visible in the *Taco1*^*mut/mut*^ (white arrows) compared with the wild-type adult mice. Representative images of haematoxylin-and-eosin-stained brains from *Taco1*^*wt/wt*^ and *Taco1*^*mut/mut*^ mice (*n*=8).

To determine if the *Taco1* mutation caused mitochondrial dysfunction, we measured the activities of all the respiratory complexes in *Taco1*^*wt/wt*^ and *Taco1*^*mut/mut*^ mice (Fig. 7a and Supplementary Fig. 6a). Both young and adult *Taco1*^*mut/mut*^ mice have significantly reduced cytochrome *c* oxidase enzyme activity in the liver and the heart (Fig. 7a and Supplementary Fig. 6a) compared with the *Taco1*^*wt/wt*^ mice, but the activities of the other respiratory complexes are not affected by the mutation in both young and adult mice. Respiration was measured in mitochondria isolated from livers and hearts of *Taco1*^*wt/wt*^ and *Taco1*^*mut/mut*^ young and adult mice and we found that the *Taco1*^*mut/mut*^ mice have reduced respiration when a substrate delivered electrons to complex IV in both tissues (Fig. 7b and Supplementary Fig. 6b). Respiration was normal in the presence of substrates delivering electrons to complex I and III, indicating that the ∼50% decrease in complex IV enzyme activity is likely not sufficient to limit the electron flux when NADH or succinate were used as substrates. Thus, loss of the TACO1 protein results in compromised formation of the terminal complex of the electron transport chain, complex IV, which compromises respiration and activity of cytochrome *c* oxidase.

### The mutant mice have motor dysfunction and heart hypertrophy

Next, we analysed the phenotypic consequences of the isolated complex IV defect caused by the *Taco1* mutation in the mice. We investigated the motor coordination, strength and balance in the *Taco1*^*wt/wt*^ and *Taco1*^*mut/mut*^ mice using hanging wire and rotarod tests. In the hanging wire test, *Taco1*^*mut/mut*^ mice moved across the wire and reached the box located at the end of the wire less often than the *Taco1*^*wt/wt*^ mice (Fig. 7c). In addition, the *Taco1*^*mut/mut*^ mice travelled less distance across the wire than the *Taco1*^*wt/wt*^ mice (Fig. 7d). *Taco1*^*wt/wt*^ mice showed improved performance on the rotarod after 4 days that was significantly decreased for *Taco1*^*mut/mut*^ mice showing that despite a normal baseline level of balance, coordination and motor function as assessed by the test, the mice are unable to improve and have motor learning deficits (Fig. 7e). At day 4, the *Taco1*^*mut/mut*^ mice performed significantly worse (*P*<0.05) than *Taco1*^*wt/wt*^ mice (Fig. 7f). Furthermore, the *Taco1*^*mut/mut*^ mice had abnormal gait and taken together with the behavioural findings this shows that the *Taco1* mutation impacts motor coordination. The young mice did not show any differences in their performance or appearance, whereas the adult *Taco1*^*mut/mut*^ mice have ruffled, greying coats compared with their age- and littermate-matched *Taco1*^*wt/wt*^ mice, consistent with premature ageing of the skin (Supplementary Fig. 6c).

Next, we investigated the effects of the mutation on cardiac function by echocardiography (Supplementary Fig. 6d). The adult *Taco1*^*mut/mut*^ mice have an increased fractional shortening, increased thickening of the left ventricular posterior wall in diastole and the left ventricular posterior wall in systole, and increased thickening of the intraventricular septum in diastole and systole compared with the *Taco1*^*wt/wt*^ mice (Supplementary Fig. 6d). These results show that the *Taco1*^*mut/mut*^ mice have cardiac dysfunction and develop hypertrophy, likely a compensatory response to the decreased activity of cytochrome *c* oxidase (Fig. 2a–d).

### *Taco1* ^*mut/mut*^ mice have visual impairment

Optokinetic drum experiments were performed to test the vision of the *Taco1*^*mut/mut*^ relative to the *Taco1*^*wt/wt*^ mice (Fig. 7g,h). The adult *Taco1*^*mut/mut*^ mice spent significantly less time tracking (following the lines on the drum with head movements; Fig. 7g) and performed significantly fewer head tracks indicating a deficiency in visuomotor behaviour (Fig. 7h). However, light/dark experiments assessing total time spent in the dark versus light showed no significant difference between *Taco1*^*wt/wt*^ and *Taco1*^*mut/mut*^ mice, indicating that the mutant mice are able to distinguish between light and dark in a similar manner to the control mice (Fig. 7i). We investigated the structure and morphology of the retina and the optic nerve using cresyl violet staining in the young and adult mice. We found that only the adult mutant mice had a reduction in the thickness of the outer plexiform layer and the inner nuclear layer indicative of retinal neuron degeneration (Fig. 7j). Measurements of the outer plexiform and inner nuclear layers revealed that their thickness was significantly reduced in the *Taco1*^*mut/mut*^ mice (Fig. 7k). Furthermore, we observed signs of atrophy in the optic nerve of adult *Taco1*^*mut/mut*^ mice compared with control mice. The former showed mild loss of the relatively large myelinated axons and increased number and size of astrocytes (Fig. 7l). Finally, Purkinje cell loss is visible in the folia of the cerebellum in adult *Taco1*^*mut/mut*^ mice compared with control mice (Fig. 7m).

## Discussion

In yeast mitochondria, translational activators are nuclear encoded proteins that regulate translation either by specifically recognizing 5′-UTRs of mitochondrial mRNAs or interacting with proteins of the small and large subunit of the ribosome to facilitate translation. Translational activators are important in restricting translation to the membrane surface by binding to the mitochondrial inner membrane and are present in limiting amounts to control the expression of their target RNAs. In some cases, translational activators have been found to bind directly or indirectly to the nascent polypeptide chains in order to guide them to the assembly machinery^20^. In mammalian mitochondria, TACO1 is the only specific translational activator identified to date^9^. We investigated the role of TACO1 and the *in vivo* molecular and pathophysiological changes caused by a homozygous missense point mutation in the *Taco1* gene in mice that causes loss of the TACO1 protein, as it has been observed for the human *TACO1* mutation that causes late-onset LS^9^. We identified that TACO1 binds to the *mt-Co1* mRNA at distinct regions and that it associates with the small ribosomal subunit and the monosome. Loss of TACO1 caused an isolated complex IV deficiency resulting in reduced mitochondrial complex activity and oxygen consumption at this site.

Complex IV is the terminal enzyme of the respiratory chain and its role is to catalyse the oxidation of cytochrome *c* by molecular oxygen to release energy, which is used to translocate protons across the inner mitochondrial membrane. Loss of TACO1 reduced the synthesis of COXI that is required, along with COXIV, to form the subcomplex S2 for the assembly of complex IV^16^ and consequently resulted in significantly decreased cytochrome *c* oxidase abundance and activity. In addition, the loss of TACO1 and consequent reduced abundance of COXI followed by COXII decrease resulted in a compensatory response by decreasing nuclear encoded subunits of complex IV. It has been found that yeast translational activators control the level of mitochondrial protein synthesis and modulate the production of proteins according to the efficiency of their assembly. In this way, translational activators prevent the accumulation of unassembled subcomplexes, which may be highly deleterious to the cell^21^. Mitochondrial ribosomes in yeast require translational activators to initiate translation of mitochondrial mRNAs and in addition these activators are likely to support the positioning of ribosomes at the inner membrane to allow for polypeptide insertion into the OXPHOS machinery. We show that loss of a translational activator does not affect the stability of the *mt-Co1* mRNA, but that through the association of TACO1 with the mitochondrial ribosome, which is responsible for decoding of mRNAs, it facilitates COXI translation. Here we discover that TACO1 is an RNA-binding protein that binds to multiple adenine-guanine-rich sequences within the *mt-Co1* mRNA. The *mt-Co1* mRNA is one of the most abundant mitochondrial mRNAs^22^,^23^, however, it is not clear why it requires a dedicated translational activator or if there are other proteins or features of the mitoribosome that fulfill this role for the remaining mitochondrial mRNAs. Although TACO1 is associated with the mitoribosome, its loss does not alter the distribution of *mt-Co1* mRNA in sucrose gradients. Therefore, TACO1 must act at a stage in the translation cycle after the initial association of *mt-Co1* mRNA with the ribosome. As TACO1-binding sites are most represented at the 5′ end of the *mt-Co1* mRNA, it may be that it is involved in translation initiation or the early stages of polypeptide elongation.

The crystal structure of TACO1 revealed a distinctive three-domain architecture, where domain 1 was highly positively charged and important for RNA binding. In addition, the structure suggests flexibility of individual domains that would enable a conformational change upon RNA binding further supported by the existence of open and closed states of other DUF28 family members. Structural alignments found that proteins of the DUF28 family, exemplified by bacterial YebC proteins, displayed a very similar fold to TACO1, although interestingly there was also significant similarity between TACO1 and the archaeal and eukaryotic nuclear RNase P protein subunit Rpp30 (Supplementary Table 2). This protein has been shown to directly bind H1 RNA, the RNA subunit of the RNase P enzyme^24^, providing additional evidence that the DUF28-fold may have diverse roles in RNA metabolism. Our observation that TACO1 is an RNA-binding protein is the first description of a molecular function for the DUF28 family of proteins, which are widely distributed in eukaryotes and bacteria. In bacteria, the phylogenetic distribution of DUF28 genes closely parallels that of genes encoding components of the RuvABC complex, which are involved in resolution of Holiday junctions to enable chromosome segregation^25^, however, experimental evidence for this role is lacking. Whether this protein family is actually involved in bacterial RNA metabolism and translational control or if this is an evolutionarily new path for these proteins in the evolution of mitochondria remains to be determined.

Compromised OXPHOS activity affects tissues with high-energy demands most severely, resulting in a wide range of clinical presentations. Mutations in mtDNA and in nuclear genes encoding mitochondrial proteins cause diverse pathologies affecting specific tissues^8^ and it is particularly important to understand the underlying mechanisms that cause heterogeneic disease phenotypes. Mouse models of early-onset LS caused by cytochrome *c* oxidase deficiency have been developed previously by knocking out or knocking in a mutation in the gene *Sco2*, encoding an assembly factor of complex IV^26^, to understand the pathology of this disease. Constitutive knockout of the assembly factor gene *Sco2* was embryonic lethal, whereas a knock-in mutation caused mild pathologies in mice compared with those observed in LS patients caused by mutations in the *Sco2* gene^26^. Interestingly, a constitutive gene knockout of another assembly factor of complex IV, SURF1, was not embryonic lethal and the mice were found to have an increased lifespan and reduced brain damage after excitotoxic insults^27^. Both the *Surf1* gene knockout and the *Sco2* knock-in resulted in milder pathologies in the mice compared with those found in patients with mutations in these genes.

In our study, the mutation in the ubiquitously expressed TACO1 produces an isolated complex IV deficiency that does not cause embryonic lethality and has given us the advantage to investigate the molecular changes due to the *Taco1* mutation with age. Because the human *TACO1* mutation causes a late-onset LS and is progressive with age, we used young and adult mice and found that, similar to the human patients, the loss of TACO1 caused reduced complex IV activity and translation of COXI and the consequences of these molecular defects were apparent with age. The reduction of complex IV activity by ∼50% is similar to that found in the *Surf1* knockout and *Sco2* knock-in mice, which could explain the milder pathologies with later onset in these mouse models^26^,^27^. The *Taco1*^*mut/mut*^ mice develop adult onset signs of visual impairment, retinal degeneration, cardiohypertrophy and motor dysfunction as a result of an isolated complex IV deficiency. These defects are similar to the clinical features observed in patients homozygous for the *TACO1* mutation, making these mice a potential model of late-onset LS.

The characterization of this mouse has provided insights into the role of TACO1 in mitochondrial gene expression and the molecular defects caused by a mutation in the *Taco1* gene that may be used as a model for late-onset LS. Elucidating the roles of mitochondrial translation factors enhances our understanding of how mitoribosomes recognize, decode and translate mitochondrial mRNAs and how these processes are dysregulated in disease. Understanding the underlying molecular mechanisms by which mutations causes mitochondrial dysfunction is important for future development of tailored treatments and therapeutics for many human diseases.

## Methods

### Animals and housing

Male age- and littermate-matched (6–8 weeks ‘young' and 30 weeks ‘adult') wild-type (*Taco1*^*wt/wt*^) and homozygous mutant mice (*Taco1*^*mut/mut*^) ENU mice on a C57BL/6J background were obtained from the Australian Phenomics Facility missense mutation library. The TACO1 mice were bred onto a wild-type C57Bl/6J background for eight to ten generations. Animals were singly housed in standard cages (45 × 29 × 12 cm^3^) under a 12-h light/dark schedule (lights on 07:00 to 19:00 hours) in controlled environmental conditions of 22+2 °C and 50+10% relative humidity. Normal chow diet with physiological levels of thiamine (Rat & Mouse Chow, Specialty Foods) and water were provided *ad libitum*. All trials were conducted within the same 6 h of the light cycle. The study was approved by the Animal Ethics Committee of the UWA (AEC 03/100/526) and performed in accordance with the Principles of Laboratory Care (NHMRC Australian code for the care and use of animals for scientific purposes, 8th Edition 2013).

### Mitochondrial isolation

Mitochondria were isolated from homogenized hearts and livers and isolated by differential centrifugation as described previously^28^ with some modifications. Livers were homogenized in buffer containing 250 mM sucrose, 5 mM Tris, 1 mM EGTA, pH 7.4, with EDTA-free Complete protease inhibitor cocktail (Roche) and hearts were homogenized in 210 mM mannitol, 70 mM sucrose, 10 mM Tris, 0.1 mM EDTA, pH 7.4, containing EDTA-free Complete protease inhibitor cocktail.

### Sucrose gradient subfractionation

Sucrose gradient subfractionation was carried out as described previously^29^. Briefly, isolated mitochondria were lysed in 10 mM Tris-HCl, pH 7.4, 260 mM sucrose, 100 mM KCl, 20 mM MgCl_2_ and 1% *n*-Dodecyl β-D-maltoside (DDM) in the presence of RNasin and protease inhibitors for 30 min, the lysate centrifuged at 10,000*g* for 45 min at 4 °C, the clarified lysate was loaded on a continuous 10–30% sucrose gradient (in 10 mM Tris-HCl, pH 7.4, 100 mM KCl, 20 mM MgCl_2_ in the presence of RNasin and protease inhibitors) and centrifuged at 71,000*g* in an Optima Beckman Coulter preparative ultracentrifuge. Fractions were collected and precipitated with 30% trichloroacetic acid, washed in acetone and the entire fraction was resolved by SDS–PAGE. Protein markers of the mitochondrial ribosomal subunits were detected by immunoblotting as described below.

### Expression plasmids

Transient expression vectors were based on pcDNA3 (Invitrogen). Mouse TACO1, MRPS27 and MRPL44 were expressed fused to a C-terminal FLAG tag, or enhanced green fluorescent protein (BD Biosciences) at the C-terminus. Mitochondria targeted enhanced green fluorescent protein was used as a negative control. Plasmids were tested for expression by transfection and immunoblotting.

### Cell culture and transfections

Mouse NIH-3T3 cells (obtained from American Type Culture Collection) were cultured at 37 °C under humidified 95% air/5% CO_2_ in DMEM (Gibco, Life Technologies) containing glucose (4.5 g. l^−1^), 1 mM pyruvate, 2 mM glutamine, penicillin (100 U. ml^−1^), streptomycin sulfate (100 μg ml^−1^) and 10% fetal bovine serum. NIH-3T3 cells were plated at 60% confluence in 15 cm dishes and transfected with mouse expression plasmids in OptiMEM media (Gibco, Life Technologies). 158 ng cm^−2^ of plasmid DNA was transfected using Fugene HD (Roche). Cell incubations were carried out for up to 72 h following transfection.

### Immunoprecipitation

NIH-3T3 cells were transfected in 15 cm plates and washed in DPBS/Ca/Mg (DPBS with 1 mM CaCl_2_ and 0.5 mM MgCl_2_) and crosslinked with 2 mM dithiobis[succinimidyl propionate] at room temperature for 30 min. After quenching with 50 mM glycine and washing in DPBS/Ca/Mg, cells were lysed by sonication in lysis buffer (10 mM Tris-HCl (pH 7.5), 260 mM sucrose, 100 mM KCl, 20 mM MgCl_2_, 1% digitonin) in the presence of RNasin and protease inhibitors. Lysates were clarified by centrifugation and diluted in lysis buffer without digitonin until the final digitonin concentration was 0.2%. Diluted lysates were incubated with anti-FLAG M2 magnetic beads for 2 h at 4 °C. Beads were washed in lysis buffer with 0.1% digitonin and then in lysis buffer without digitonin. Proteins were eluted with 400 ng μl^−1^ 3x FLAG peptide and crosslinks were reversed by incubation with 100 mM dithiothreitol (DTT) at 65 °C for 20 min.

### RNA isolation and northern blotting

RNA was isolated from mitochondria using the miRNeasy Mini kit (Qiagen) incorporating an on-column RNase-free DNase digestion to remove all DNA. RNA (5 μg) was resolved on 1.2% agarose formaldehyde gels, then transferred to 0.45 μm Hybond-N^+^ nitrocellulose membrane (GE Lifesciences) and hybridized with biotinylated oligonucleotide probes specific to mouse mitochondrial mRNAs, rRNAs and tRNAs. The hybridizations were carried out overnight at 50 °C in 5x SSC, 20 mM Na_2_HPO_4_, 7% SDS and 100 μg ml^−1^ heparin, followed by washing. The signal was detected using either streptavidin-linked horseradish peroxidase or streptavidin-linked infrared antibody (diluted 1:2,000 in 3x SSC, 5% SDS, 25 mM Na_2_HPO_4_, pH 7.5) by enhanced chemiluminescence (GE Lifesciences) or using an Odyssey Infrared Imaging System (Li-Cor), respectively.

### Preparation of fluorescently labelled RNAs

Overlapping 200 bp fragments corresponding to the mouse *mt-Co1* mRNA were synthesized from DNA oligonucleotides and each fragment was cloned into pMA-7-Ar (GeneArt, Life Technologies), flanked at the 5′ end by a T7 promoter sequence and at the 3′ end by an *Xma*I recognition site. Plasmids were linearized by *Xma*I digestion and fluorescently labelled RNAs were synthesized via direct incorporation of aminoallyl-UTP-ATTO-680 (Jena Bioscience) during *in vitro* transcription with the MAXIscript T7 kit and purified using NucAway spin columns (Ambion, Life Technologies).

### RNA electrophoretic mobility shift assays

Purified TACO1 protein (at equimolar to eightfold in excess concentration relative to the probe) was incubated at room temperature for 30 min with 100 ng μl^−1^ ATTO-680-labelled RNAs in 10 mM HEPES (pH 8.0), 1 mM EDTA, 50 mM KCl, 2 mM DTT, 0.1 mg ml^−1^ fatty acid-free bovine serum albumin (BSA) and 0.02% Tween-20. Reactions were analysed by 10% PAGE in Tris-acetate-EDTA (TAE) and fluorescence was detected using an Odyssey Infrared Imaging System (Li-Cor).

### RNA microarray

A custom RNA microarray was produced and analysed by LC Sciences. The microarray consisted of 36-nt single-stranded variable RNA sequences and scanned through the region of mouse mtDNA (EF108336.1) between positions 4,932 and 8,622 in 18-nt increments, a selection of control RNAs were also scanned in 18-nt increments (*Rpph1*, NR_002142.2, nt 1–324; *Rn5S*, NR_030686.1, nt 1–121; *Trm1*, tM(CAT)M10 from Coughlin *et al.*^30^, nt 1–80; *Actb*, NM_007393.3, nt 708–1283), and the *mt-Co1* region between positions 5,016 and 7,011 in 1-nt increments. The freshly synthesized microarray chip was filled with washing buffer (10 mM HEPES, 100 mM NaCl, 50 mM KCl, 1 mM EDTA, 2 mM DTT, 0.1 mg ml^−1^ BSA, 0.02% Tween-20, pH 7.4), heated to 55 °C for 5 min and slowly cooled to 30 °C at −2 °C min^−1^. Blocking buffer (10 mM HEPES, 100 mM NaCl, 50 mM KCl, 1 mM EDTA, 2 mM DTT, 10 mg ml^−1^ BSA, 0.02% Tween-20, pH 7.4) was circulated inside the microarray chip for 2 h at 25 °C and 10 nM purified mouse TACO1 was applied to the microarray for 2 h at 25 °C in washing buffer. The microarray was then washed with the same buffer without protein and Alexa-647-labelled antihexahistidine antibody applied to the chip using the same conditions as TACO1. The microarray was washed at 25 °C in washing buffer and then scanned at 635 nm.

### Immunoblotting

Specific proteins were detected using rabbit polyclonal antibodies against: MRPL44 (16394-1-AP), MRPL23 (11706-1-AP), TACO1 (21147-1-AP), MRPS35 (16457-1-AP), MRPS16 (16735-1-AP) (Proteintech, diluted 1:1,000), MRPS34 (Sigma HPA042112, diluted 1:1,000), and mouse monoclonal antibodies against: β-actin (8226), porin (14734), NDUFA9 (14713), complex II (14715), complex III (14745), COXI (14705), COXII (198286), COXIV (14744) and complex V subunit a (14748; Abcam, diluted 1:1,000), in Odyssey Blocking Buffer (Li-Cor). IR Dye 800CW Goat Anti-Rabbit IgG or IRDye 680LT Goat Anti-Mouse IgG (Li-Cor) secondary antibodies were used and the immunoblots were visualized using an Odyssey Infrared Imaging System (Li-Cor). Examples of uncropped blots are shown in the Supplementary Fig. 8. Tissue-specific analysis was performed using a Proteintech mouse tissue blot (Cat. No M10005).

### Mitochondrial protein synthesis

Mitochondria were isolated from hearts and livers of *Taco1*^*wt/wt*^ and *Taco1*^*mut/mut*^ mice as described above and *de novo* protein synthesis was analysed as described before^29^. Briefly, 500 μg mitochondria were incubated in 750 μl translation buffer (100 mM mannitol, 10 mM sodium succinate, 80 mM KCl, 5 mM MgCl_2_, 1 mM KPi, 25 mM HEPES, pH 7.4, 5 mM ATP, 20 μM GTP, 6 mM creatine phosphate, 60 μg ml^−1^ creatine kinase and 60 μg ml^−1^ of all amino acids except methionine). Mitochondria were supplemented with 150 μCi of ^35^S methionine (PerkinElmer) for 60 min at 37 °C. After labelling, mitochondria were washed in translation buffer and suspended in RIPA lysis buffer. Protein concentration was measured and 50 μg of mitochondrial protein was resolved by SDS–PAGE and visualized by autoradiography.

### Blue native PAGE electrophoresis

BN-PAGE was carried out using isolated mitochondria from livers and hearts of *Taco1*^*wt/wt*^ and *Taco1*^*mut/mut*^ using Invitrogen pre-cast gels according to the manufacturer's instructions and as described before^31^. Samples resolved by BN-PAGE were analysed by transferring to polyvinylidene difluoride and immunoblotting against the respiratory complexes.

### Complex enzyme assays

Enzyme assays were carried out in a 1-ml cuvette at 30 °C using a PerkinElmer lambda 35 dual beam spectrophotometer. Citrate synthase was assayed by standard procedures. Complex I was assayed as the rotenone-sensitive rate of NADH reduction of CoQ1, complex II by the thenoyltrifluoroacetone-sensitive rate of succinate reduction of 2,6-dichlorophenolindophenol. Combined complex II and complex III activity was assayed as the myxothiazol- sensitive rate of reduction of ferricytochrome *c* by succinate. Complex III was measured as the myxothiazol-sensitive rate of reduction of ferricytochrome *c* by CoQ2, complex IV was measured as the cyanide-sensitive oxidation of ferrocytochrome *c* and complex V was assayed as the oligomycin-sensitive rate of ATP hydrolysis, measured by coupling ADP production to NADH oxidation by a linked enzyme assay. Briefly, ATP hydrolysed to ADP by complex V was reconverted to ATP through reaction with phosphoenolpyruvate catalysed by pyruvate kinase, with the pyruvate produced coupled to NADH oxidation by lactate dehydrogenase as described in ref. 32.

### Respiration

Mitochondrial respiration was evaluated as O_2_ consumption in isolated mitochondria from the heart and the liver of *Taco1*^*wt/wt*^ and *Taco1*^*mut/mut*^ according to Kuznetsov *et al.*^33^. Mitochondria were supplemented with substrates for either complex I (10 mM glutamate/malate, Sigma), II (10 mM succinate, Sigma) or IV (1 mM TMPD/1 mM ascorbate, Sigma). After addition of 1 mM adenosine diphosphate (ADP, Sigma) to the recording chamber, State 3 respiration activity was measured. ADP-independent respiration activity (State 4) was monitored after addition of oligomycin (2 μg ml^−1^, Sigma).

### Protein expression and purification

The coding sequence of mouse TACO1, lacking its N-terminal mitochondria targeting sequence, (amino acids 27–294, NP_081622.1) was cloned into pETM30 and expressed as a fusion to an N-terminal His tag and glutathione S-transferase in *Escherichia coli* ER2566 cells (New England Biolabs). Wild-type and mutant proteins (Mutagenex) were purified using HIS-Select Nickel Affinity Gel according to the manufacturer's instructions (Sigma-Aldrich). The eluted protein was dialysed against 25 mM Tris-HCl (pH 7.4), 0.2 M NaCl, 0.5 mM EDTA, 2 mM DTT, 10% glycerol and further purified by gel filtration using an ÄKTAexplorer system (GE) with a Superdex 200 10/300 column with a total bed volume of 120 ml and concentrated using Vivaspin concentrators (GE) before use in RNA-binding assays. Protein concentration was determined by the bicichroninic acid assay using BSA as a standard.

Protein used for crystallization was purified as above with the following modifications: During dialysis in H-0.05, the fusion tag was removed by Tobacco Etch Virus at a protease/protein molar ratio of 1:50 followed by purification on a heparin column equilibrated in H-0.05 and eluted with H-0.3 prior to gel filtration.

### Crystallization and structure solution

Crystals were grown at 23 °C by the sitting drop vapour diffusion method by mixing 1.5 μl TACO1 (4 mg ml^−1^) with an equal volume of reservoir solution (200 mM ammonium nitrate, 160 mM sodium thiocyanate, 10% PEG 400, and 20% PEG 3350). A single-wavelength anomalous dispersion and a native data set were collected at the K-edge of selenium at beam line ID30A-3 (ESRF). The X-ray diffraction data (Supplementary Table 1) were processed with XDS^34^, and the structure was solved with PHENIX^35^. An initial model built by Phenix was used as a starting model for auto tracing by ARP/wARP^36^. Several rounds of manual building in COOT^37^ interspersed with refinement in PHENIX^35^ and Buster^38^ rendered a final model (Table 1) that had no Ramachandran outliers as assessed by MOLPROBITY^39^. A representative portion of the electron density of the final TACO1 model is shown as a stereo image in Supplementary Fig. 7. Figures were prepared with Pymol (www.pymol.org).

### Behavioural and motor testing

*Light–dark exploration test*. A light–dark tunnel was used to investigate locomotor activity and light sensitivity^40^. The 2-m-long enclosed perspex tunnel (∼15 × 15 cm^2^ square) was divided into a light (transparent) half and a dark (opaque) half. Each mouse was placed at the farthest end of the light half and the tunnel was sealed. Over the course of 10 min, the number of times the mouse crossed from the light to the dark side was recorded, as well as the total amount of time the mouse spent in the light.

*Optomotor response*. The mouse visual response to moving stimuli was investigated using an optokinetic drum fitted with a moving stimulus pattern of black and white stripes of 1 cm width^41^. The drum comprised a motorized Perspex cylinder of 30 cm diameter, which rotated around a 13-cm diameter central pedestal, with a rotational speed of 2 revolutions per minute. Mice were placed in a non-reflective transparent cylindrical container on the central pedestal. After a 2-min acclimatization period, the mouse was video recorded with the drum rotating clockwise for 2 min and then anticlockwise for 2 min, with a 2-min rest period in between. The video was analysed to record the number of tracking movements and the total time spent tracking in the 2-min period. There were not significant differences between clockwise and anticlockwise directions so an average was taken for each mouse.

*Hanging wire test*. To test for neuromuscular abnormalities^42^, mice were placed (front paws only) in the middle of a 1-mm diameter horizontal wire 50 cm above the ground. The wire was 1 m long with a wooden platform that the mice could climb onto at each end. Each mouse was tested four times on 4 consecutive days. On each day the time taken to complete the task and distance travelled were recorded. The task was stopped if the mouse fell off the wire (judged unsuccessful) or after a maximum of 1 min 30 s if the mouse had not yet reached the platform.

*Rotarod test*. Tests for muscular coordination and strength were determined by performance on an accelerating rotarod. Mice were placed on a rotating drum that gradually accelerated from 2 to 40 r.p.m. during a 5-min test. The results of three replicates of each tested were averaged and repeated for daily for 4 consecutive days to measure baseline skill and motor learning ability. Data were analysed using a two-tailed, unpaired, unequal variance Student's *t*-test.

### Echocardiography

Echocardiographic studies to measure left ventricular function were performed on mice under light methoxyflurane anaesthesia with the use of an i13L probe on a Vivid 7 Dimension (GE Healthcare). Echocardiographic measurements were taken on M-mode in triplicate from each mouse. The quantitative measurements represent the average of 30-week-old *Taco1*^*wt/wt*^ (*n*=5) and litter-mate matched 30-week-old *Taco1*^*mut/mut*^ (*n*=5) mice. M-mode recordings were made at a sweep speed of 200 mm s^−1^. Measurements of left ventricular end diastolic diameter (LVEDD), left ventricular end systolic diameter (LVESD), fractional shortening, left ventricular posterior wall in diastole, left ventricular posterior wall in systole, intraventricular septum in diastole and intraventricular septum in systole were made. Fractional shortening was calculated by the formula [(LVEDD-LVESD)/EDD] × 100.

### Histology

Mice were anaesthetized with sodium pentobarbital (150 mg kg^−1^, i.p.) and perfused transcardially with 4% paraformaldehyde in 0.1 M PBS, pH 7.4, and the brains and eyes stored in 30% sucrose. Sections were cut using a cryostat and transferred to slides. Slides were washed in distilled H_2_O (dH_2_O) for 2 min then heated to 50 °C in 5% acetic acid 95% ethanol solution. The slides were washed in dH_2_O and then incubated in cresyl stain (5% cresyl acetate, 2% sodium acetate anhydrous in dH_2_O) at 50 °C for 8 min. Slides were incubated for 11 min at room temperature in 5% acetic acid, 95% ethanol solution and then washed three times in 100% ethanol for 2 min followed by two 2 min wash in xylene. Coverslips were attached using Entellen and images were acquired using a Nikon Ti Eclipse inverted microscope using a Nikon × 20 objective.

### Data availability

All other data that support the findings of this study are available from the corresponding author upon reasonable request.

## Additional information

**Accession codes:** Atomic coordinates and structure factors for TACO1 have been deposited under the accession code 5EKZ in the Protein Data Bank.

**How to cite this article:** Richman, T. R. *et al.* Loss of the RNA-binding protein TACO1 causes late-onset mitochondrial dysfunction in mice. *Nat. Commun.* 7:11884 doi: 10.1038/ncomms11884 (2016).

## Supplementary Material

Supplementary Information Supplementary Figures 1-8, Supplementary Tables 1-2, Supplementary Methods and Supplementary References.
