# Supplementary material for: Loss of the RNA-binding protein TACO1 causes late-onset mitochondrial dysfunction in mice
Source: Nat Commun. 2016 Jun 20;7:11884. doi: 10.1038/ncomms11884 (PMC4915168; doi:10.1038/ncomms11884)
Supplement: Supplementary Information — Supplementary Figures 1-8, Supplementary Tables 1-2, Supplementary Methods and Supplementary References. [file ncomms11884-s1.pdf]

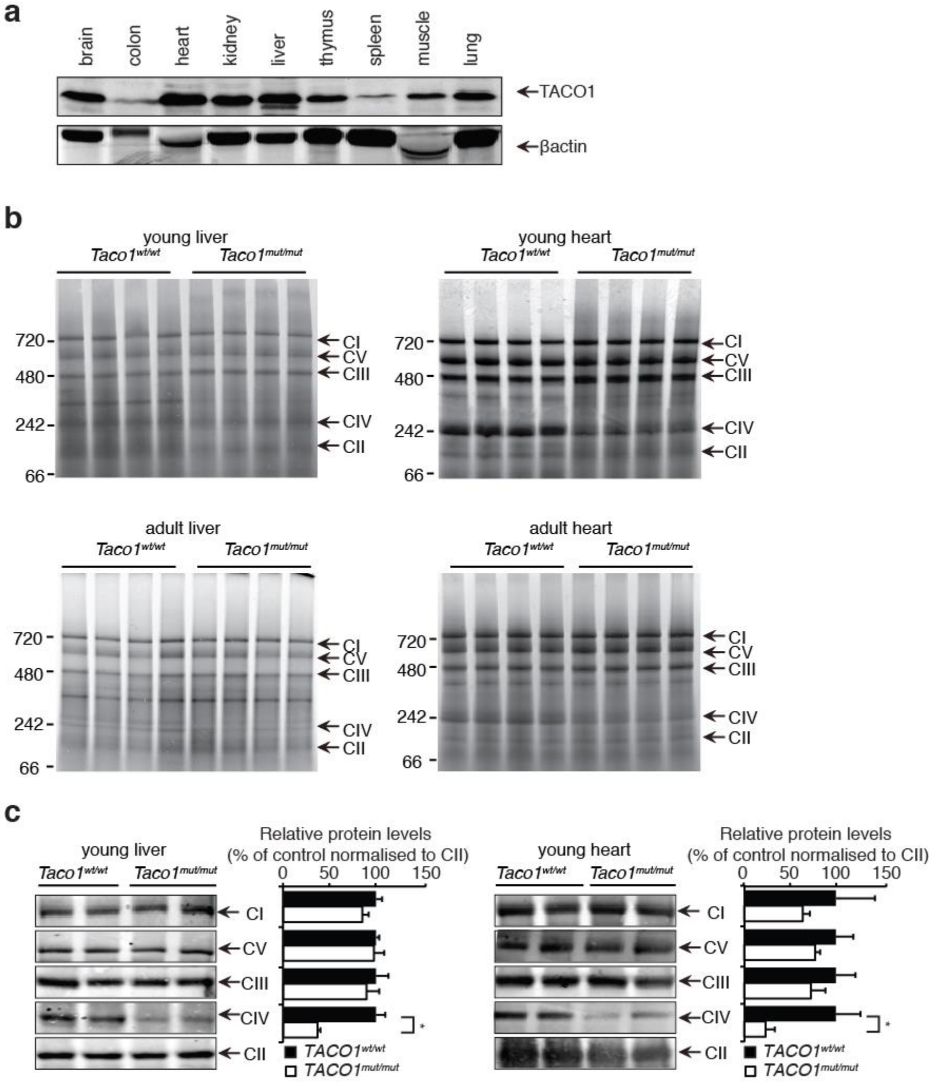

Supplementary Figure 1. Related to Figure 1 and Figure 2 | Distribution of TACO1 in mouse tissues and the effects of TACO1 loss on Complex IV biogenesis.

(**a**) Distribution of the TACO1 protein in a range of tissues from wild type mice was determined by immunoblotting.

(**b**) Mitochondrial proteins (75 µg) isolated from liver and heart *Taco1*$^{mut/mut}$ and *Taco1*$^{wt/wt}$ young and adult mice were resolved by BN-PAGE and stained with Coomassie Brilliant Blue.

(**c**) Mitochondria isolated from livers and hearts of young (50 µg) *Taco1*$^{mut/mut}$ and *Taco1*$^{wt/wt}$ mice were analyzed by BN-PAGE and immunoblotting. Specific antibodies representing proteins of each of the mitochondrial complexes were used to compare abundance of protein in the *Taco1*$^{wt/wt}$ and *Taco1*$^{mut/mut}$ mice. The data are representative of results obtained from 8 mice from each strain and at least three independent biological experiments. *, $p < 0.05$ compared with control treatments by a 2-tailed paired Student's t test.

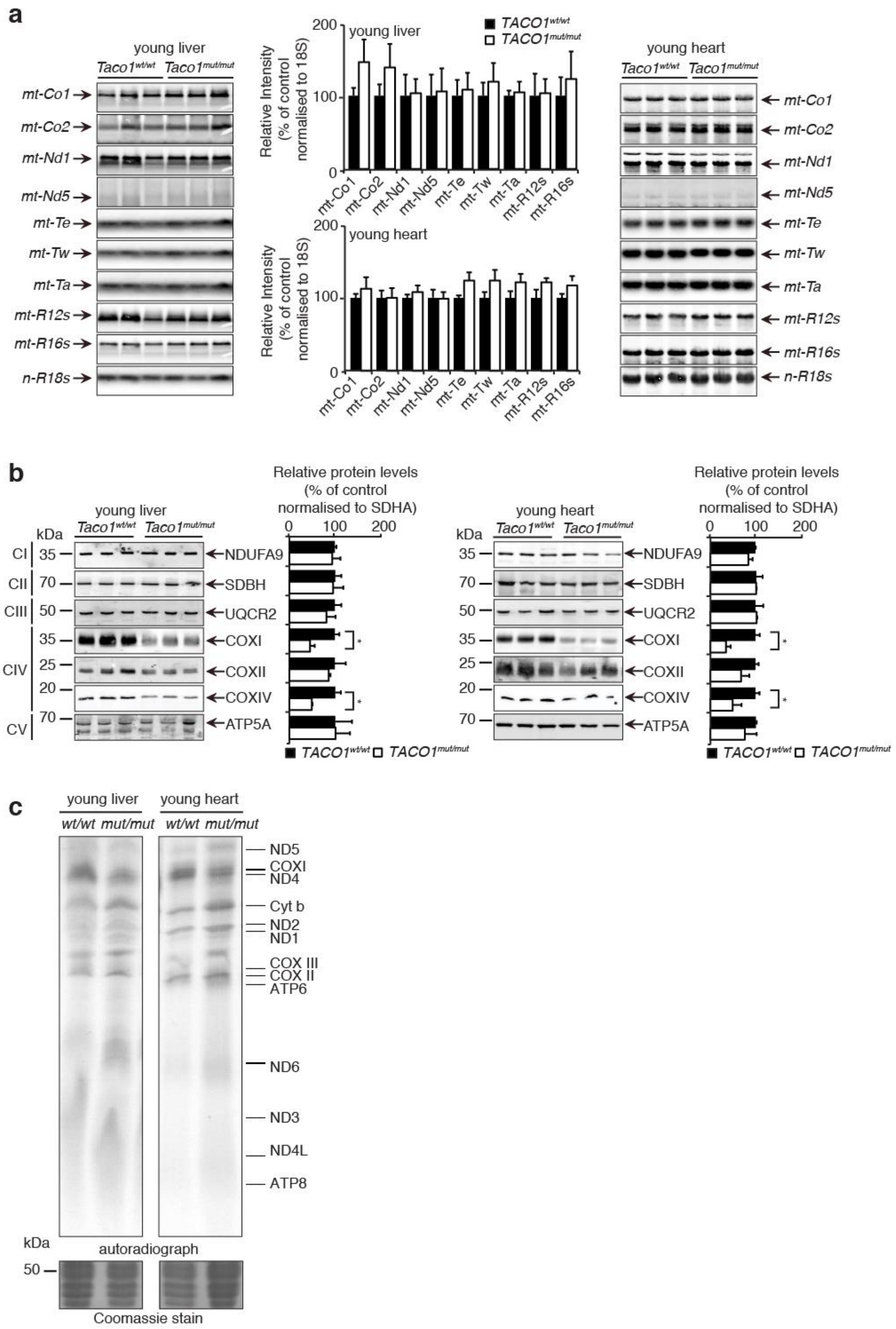

Supplementary Figure 2. Related to Figure 2 | Loss of the TACO1 protein results in reduced steady state levels of Complex IV polypeptides, decreased COXI protein synthesis and decreased Complex IV activity in young *Taco1*$^{mut/mut}$ mice**.**

(**a**) The *Taco1* mutation does not affect mitochondrial RNA metabolism. The abundance of mature mitochondrial transcripts in mitochondria isolated from young *Taco1*$^{wt/wt}$ and *Taco1*$^{mut/mut}$ livers and hearts was analyzed by northern blotting. 18S rRNA was used as a loading control. The data are representative of results obtained from at least 8 mice from each strain and three independent biological experiments. *, $p < 0.05$ compared with control treatments by a 2-tailed paired Student's t test.

(**b**) Mitochondrial proteins (20 µg) from young *Taco1*$^{wt/wt}$ and *Taco1*$^{mut/mut}$ liver and heart were resolved on SDS-PAGE gels and immunoblotted against antibodies to investigate the steady state levels of nuclear and mitochondrial-encoded proteins. Porin was used as a loading control. Representative blots are shown of three independent biological experiments. *, $p < 0.05$ compared with control treatments by a 2-tailed paired Student's t test.
(**c**) Protein synthesis in heart of young *Taco1*$^{wt/wt}$ and *Taco1*$^{mut/mut}$ was measured by pulse incorporation of ^{35}S-labelled methionine and cysteine. Equal amounts of mitochondrial protein (50 µg) were separated by SDS–PAGE, stained using Coomassie Brilliant Blue to show equal loading and visualized by autoradiography. Representative gels are shown of three independent biological experiments.

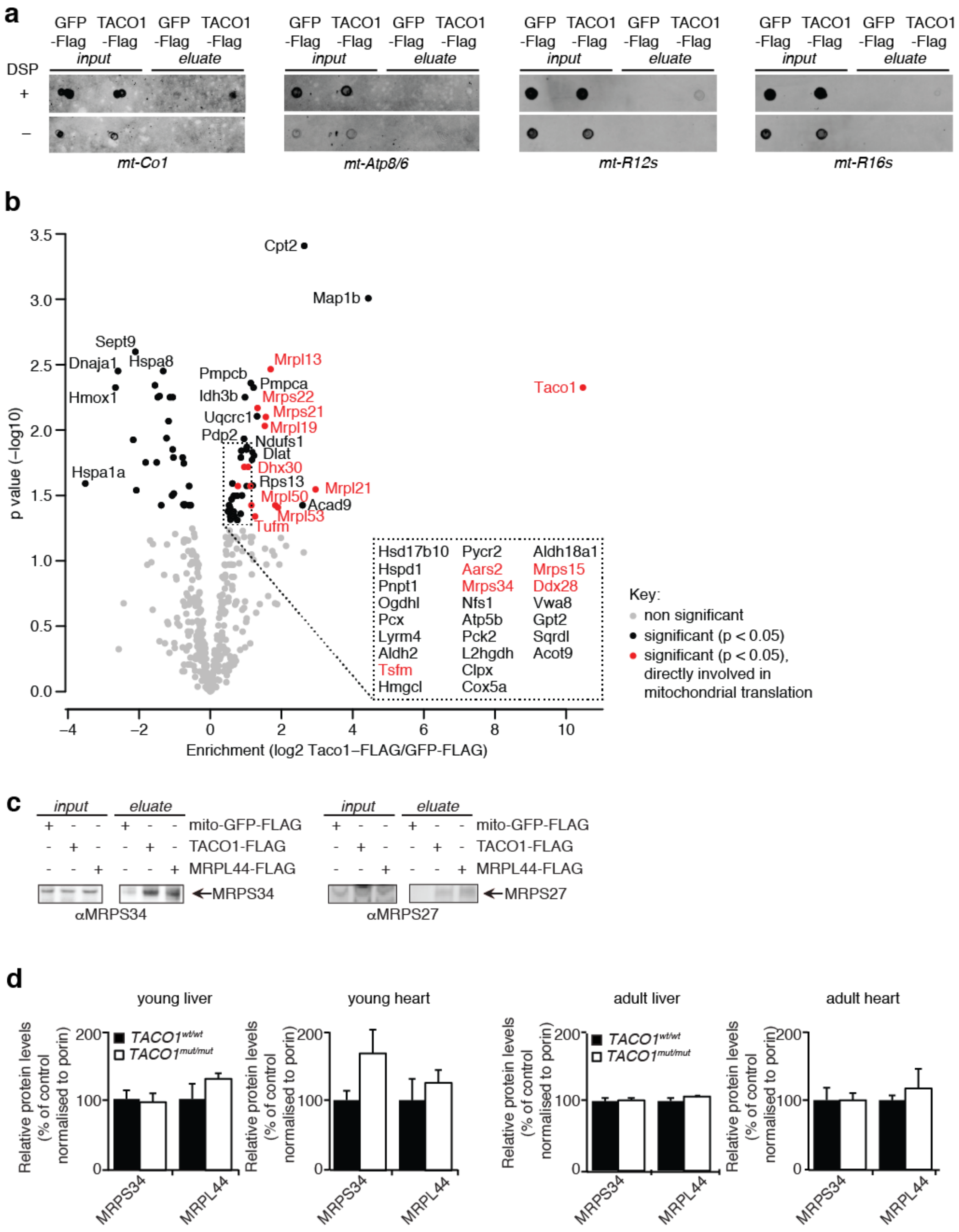

Supplementary Figure 3. Related to Figure 4 | TACO1 transiently associates with *mt-Co1* mRNA.
(**a**) Dot blot of input and immunoprecipitated RNA from TACO1-FLAG or mito-EGFP-FLAG expressing NIH-3T3 cells. The blot was probed for *mt-Co1*, *mt-Atp8/6*, 12S rRNA and 16S rRNA.
(**b**) Identification of proteins associated with TACO1 by mass spectrometry. TACO1-FLAG or control GFP-FLAG proteins were isolated from NIH-3T3 cells by crosslinking followed by immunoprecipitation and associated proteins were analysed by mass spectrometry-based label-free quantitative proteomics. Proteins where the adjusted p value was < 0.05 are shown in black. Proteins that were not significantly enriched, according to an adjusted p value of < 0.05, are shown in grey. Proteins that have been shown to be directly involved in mitochondrial translation, where the adjusted p value was < 0.05, are shown in red. Note that a number of proteins that very likely influence translation have not been highlighted (Hsd17b10 is a subunit of the mitochondrial RNase P complex,

required to produce tRNAs[1,2]; Dhx30 is found in mitochondrial RNA processing granules that are centres for mitochondrial ribosomal biogenesis[3]; Pnpt1 is part of the mitochondrial RNA degrading complex[4]; Idh3b has been identified as a mitochondrial RNA-binding protein[5]). Enrichment values are an average of three independent biological experiments.

(**c**) Immunoblots of mitochondrial ribosomal proteins immunoprecipitated TACO-FLAG, MRPL44-FLAG or mito-EGFP-FLAG expressing NIH-3T3 cells. The blots were probed for MRPS34 and MRPL27.

(**d**) Quantification of the mitochondrial ribosomal protein immunoblots shown in Fig. 4c.

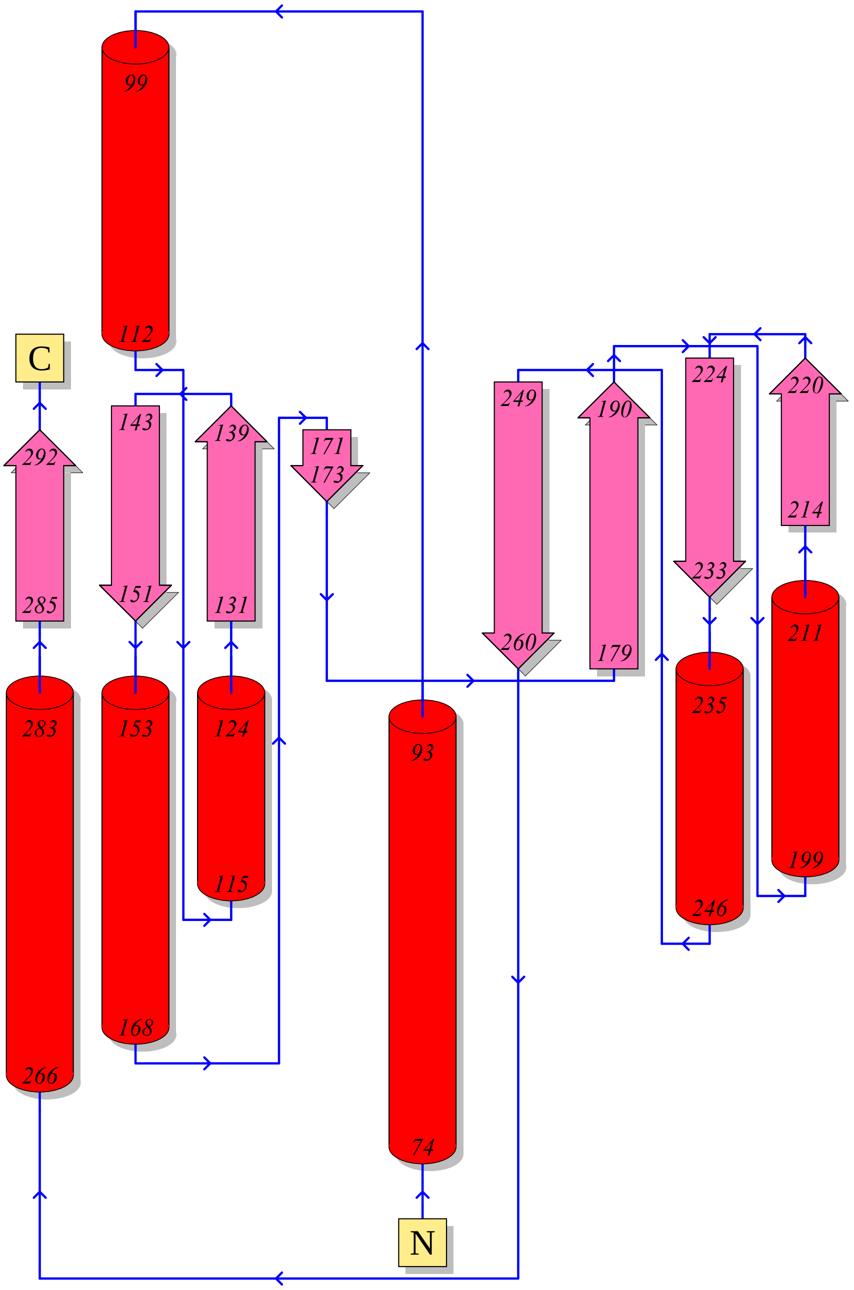

Supplementary Figure 4. Related to Figure 5 | Topology diagram of TACO1. α-helices are represented by red cylinders and β-strands by pink arrows. The beginning and end of each secondary structure element is indicated.

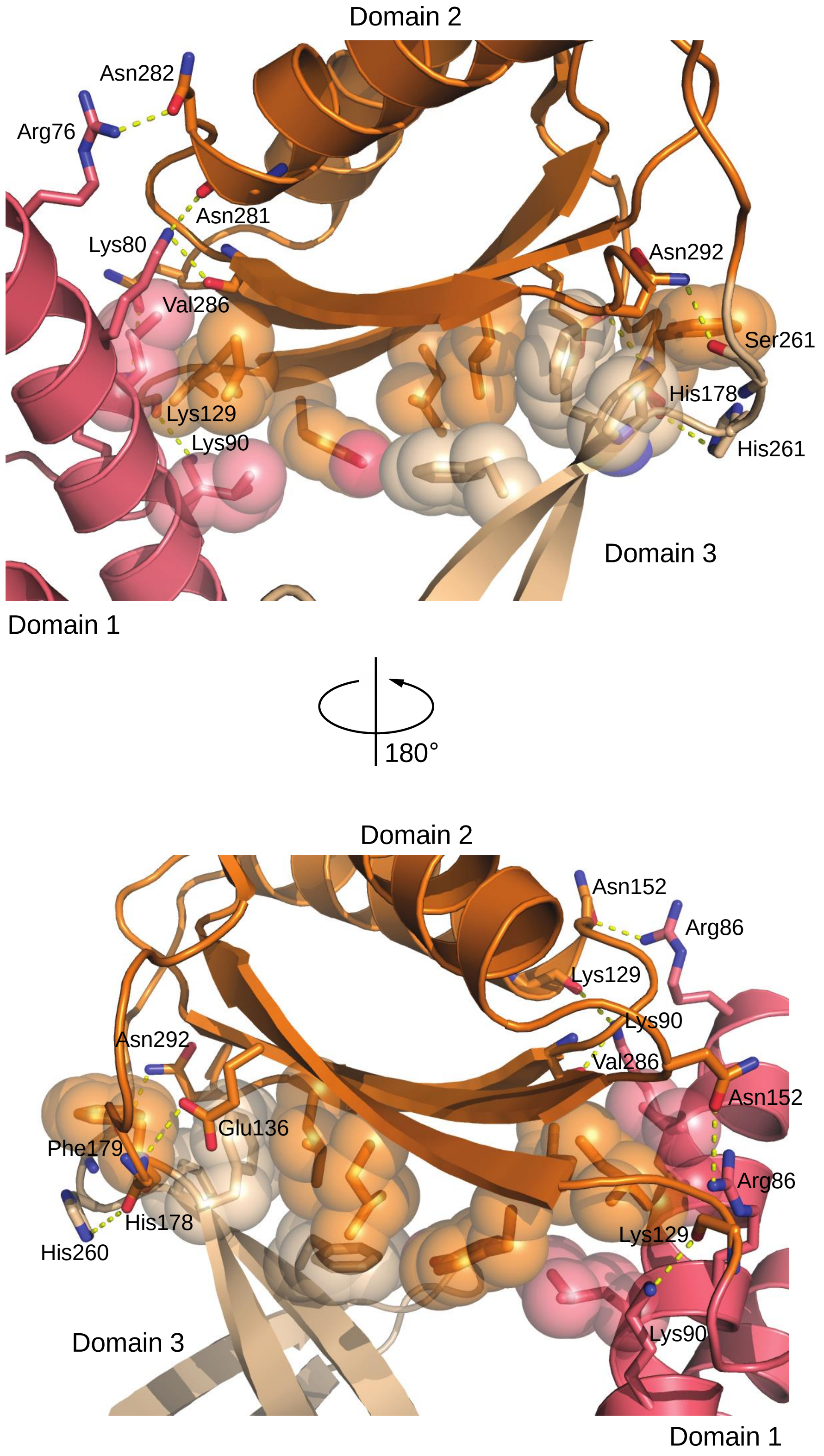

Supplementary Figure 5. Related to Figure 5 | Interface between domains. Interacting polar residues are shown as sticks and hydrophobic residues as spheres.

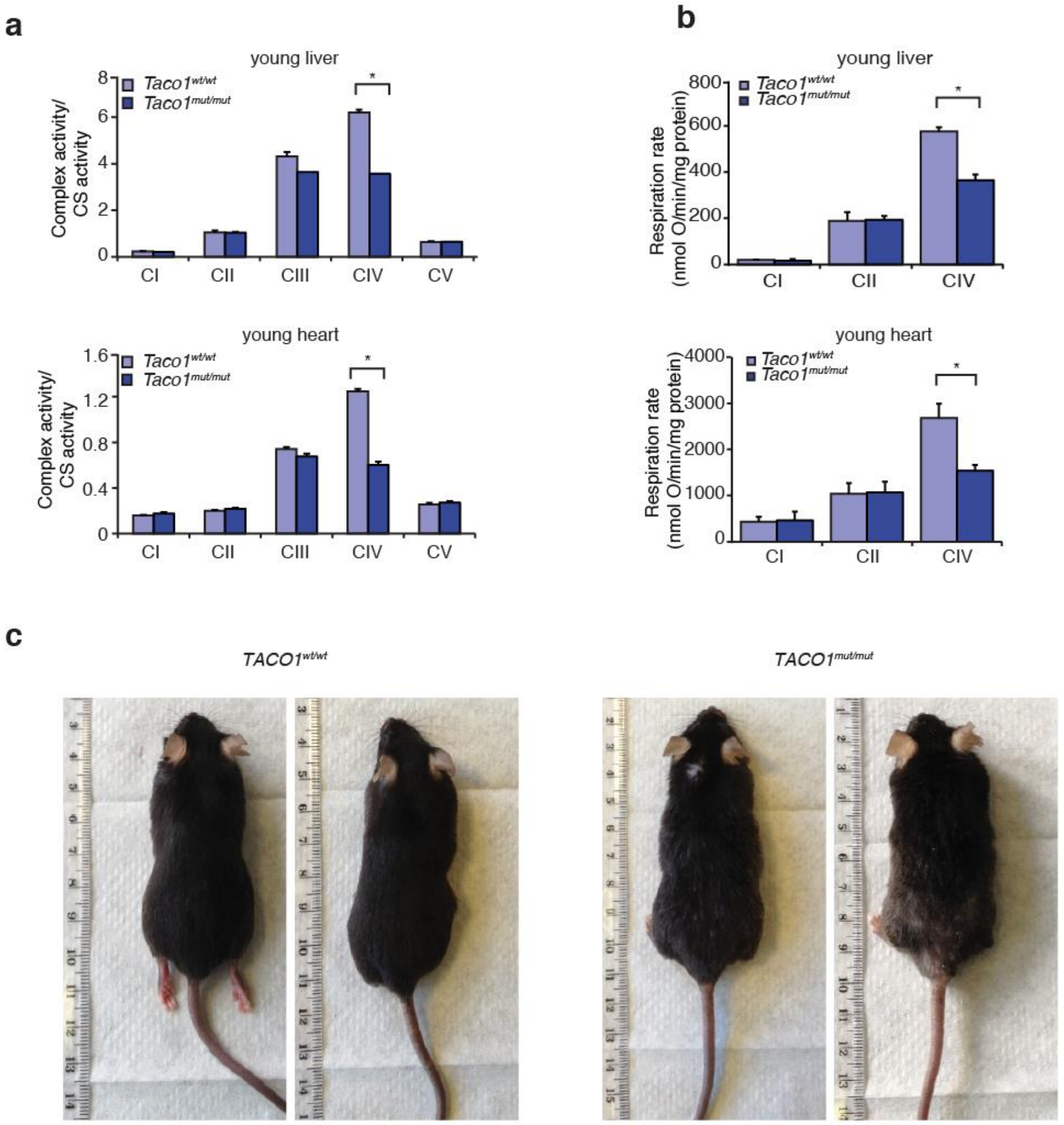

d

	LVEDD (mm)	LVESD (mm)	FS (%)	LVDPW (mm)	LVSPW (mm)	IVDS (mm)	IVSS (mm)	HR (bpm)
Taco1$^{wt/wt}$	3.41 ±0.17	2.16 ±0.20	37.31 ±2.56	0.85 ±0.02	0.96 ±0.03	0.86 ±0.01	0.88 ±0.03	524 ±15
Taco1$^{mut/mut}$	3.53 ±0.06	1.97 ±0.06	44.15* ±1.31	0.94* ±0.03	1.17* ±0.04	0.99* ±0.03	1.00* ±0.02	564 ±26

Supplementary Figure 6. Related to Figure 7 | Adult *Taco1*$^{mut/mut}$ mice have isolated cytochrome c deficiency and show signs of ageing and cardiohypertrophy by echocardiography.

(**a**) Respiratory complex activities were measured spectrophotometrically and normalized to citrate synthase activity in mitochondria isolated from livers and hearts of young *Taco1*$^{wt/wt}$ (n=8) and *Taco1*$^{mut/mut}$ mice (n=8). Data are means ± SEM of three-four separate experiments; *, $p < 0.05$ compared with control treatments by a 2-tailed paired Student's *t* test.

(**b**) Loss of the TACO1 protein causes reduced respiration at Complex IV in *Taco1*$^{mut/mut}$ mice. State 3 respiration was measured in mitochondria isolated from hearts and livers of *Taco1*$^{wt/wt}$ and *Taco1*$^{mut/mut}$ mice using an OROBOROS oxygen electrode. Data are means ± SEM of three-four separate experiments; *, $p < 0.05$ compared with control treatments by a 2-tailed paired Student's *t* test.

(**c**) Representative images of *Taco1*$^{wt/wt}$ and *Taco1*$^{mut/mut}$ mice. The *Taco1*$^{mut/mut}$ mice have a ruffled and aged appearance.

(**d**) Echocardiographic parameters of *Taco1*$^{wt/wt}$ (n=5) and *Taco1*$^{mut/mut}$ (n=5) mice. LVEDD, left ventricular end diastolic diameter; LVESD, left ventricular end systolic diameter; FS, fractional

shortening; LVDPW, left ventricular posterior wall in diastole; LVSPW, left ventricular posterior wall in systole; IVDS, intraventricular septum in diastole; IVSS, intraventricular septum in systole. Values are means ± standard error. *$p<0.05$ compared with *Taco1*$^{wt/wt}$.

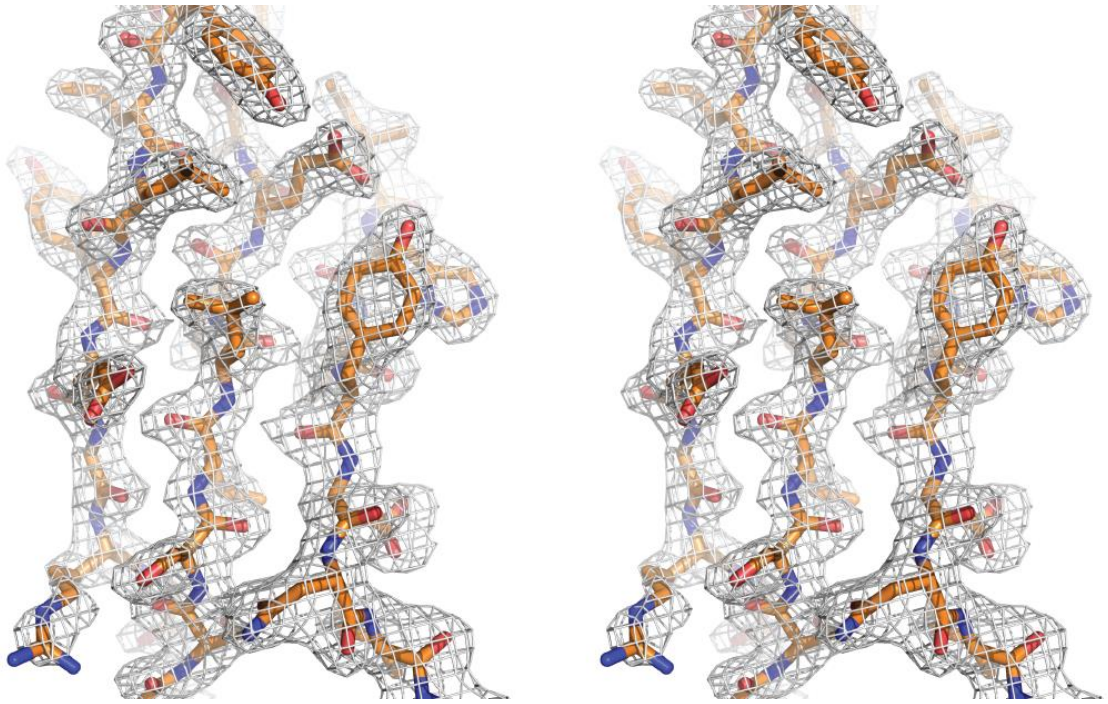

Supplementary Figure 7| Stereo image of electron density for TACO1.
Stereo image showing the final weighted $2F_O$-F_C electron density map of a representative section of TACO1 contoured at 1.5 σ.

Supplementary Figure 8 | Supplementary blots.

Scans of blots related to Figure 1 and Figure 2

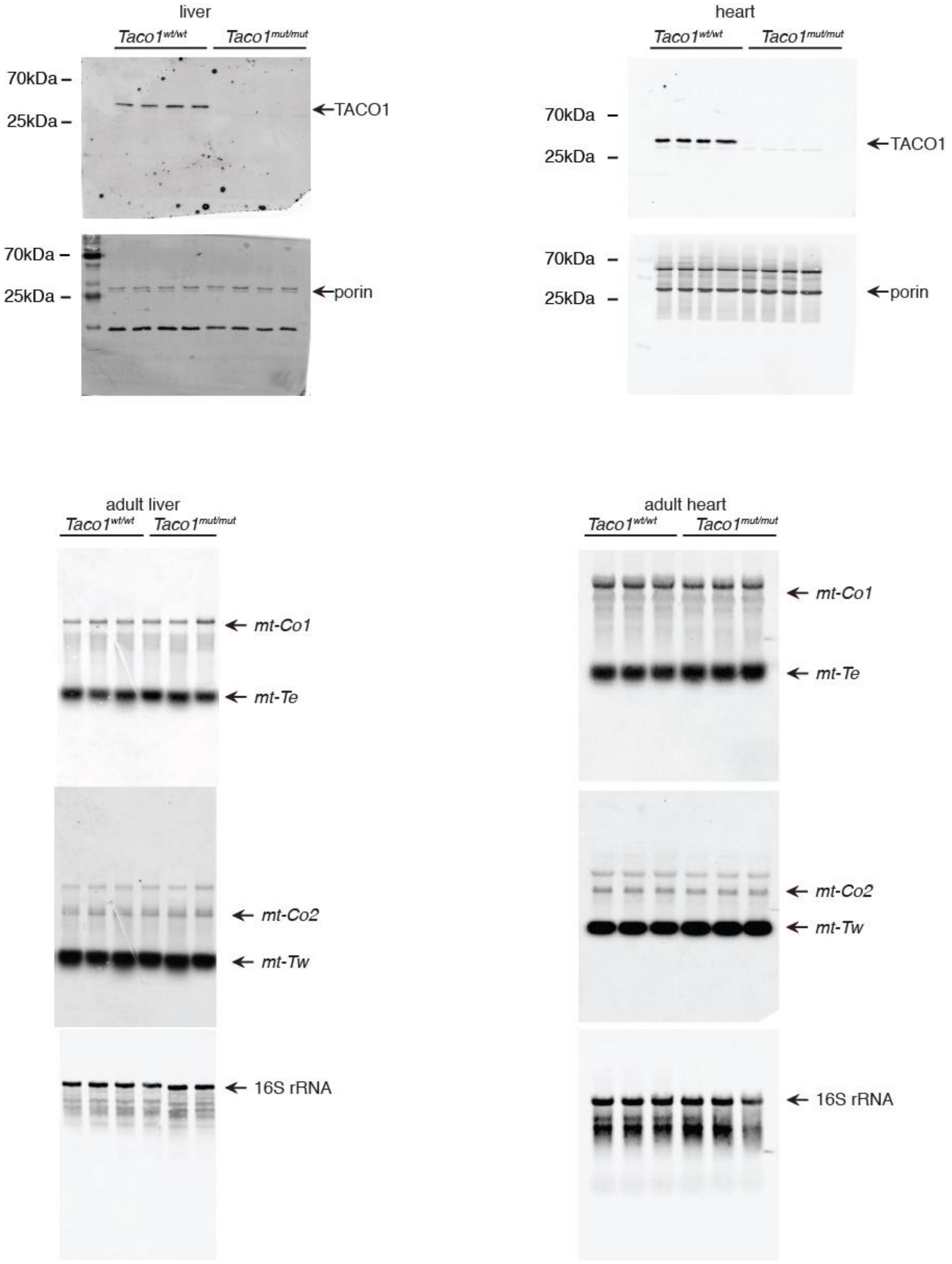

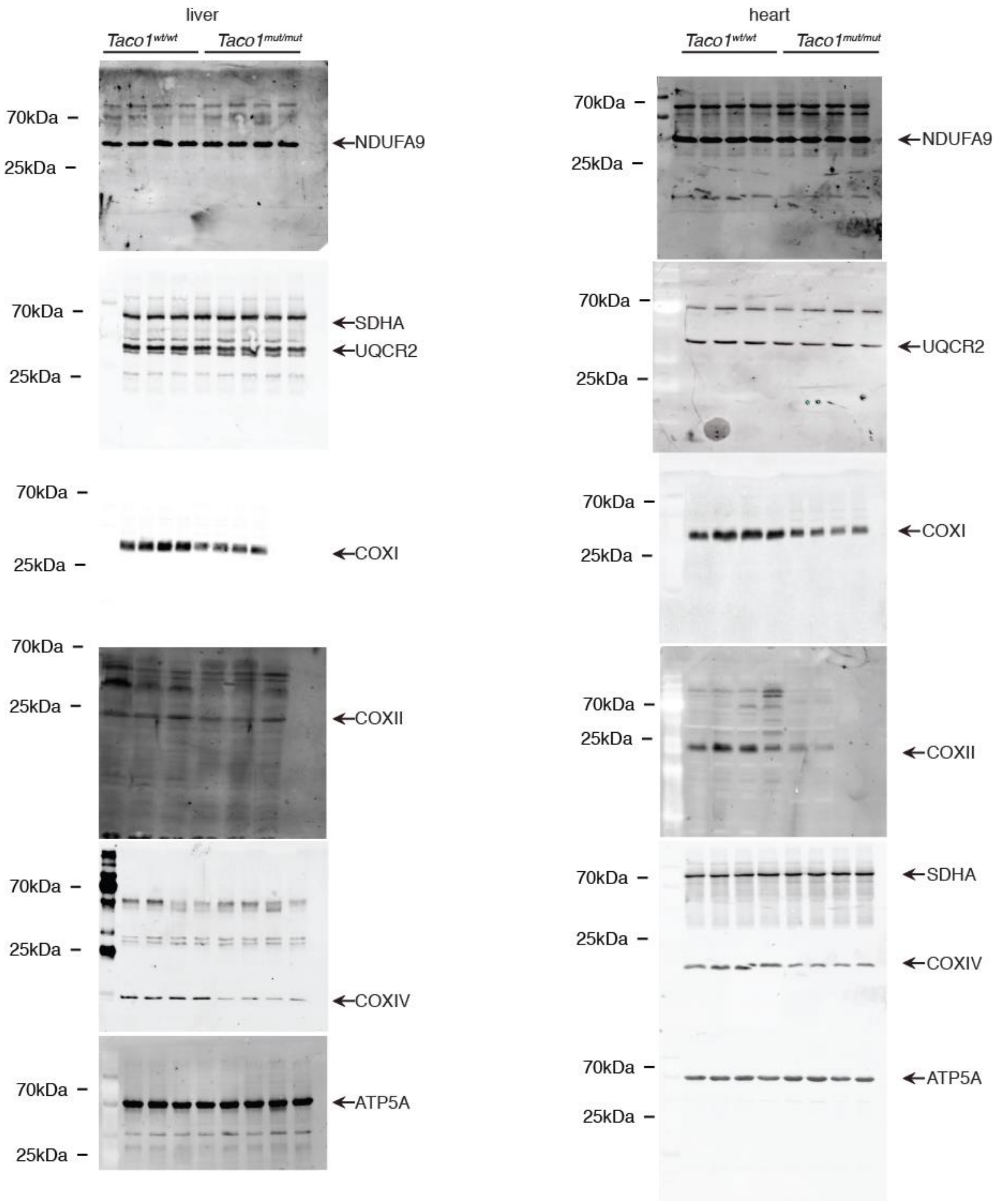

liver
Taco1wt/wt
Taco1mut/mut
70kDa
25kDa
NDUFA9
SDHA
UQCR2
COXI
COXII
COXIV
ATP5A
heart

Scans of blots related to Figure 4

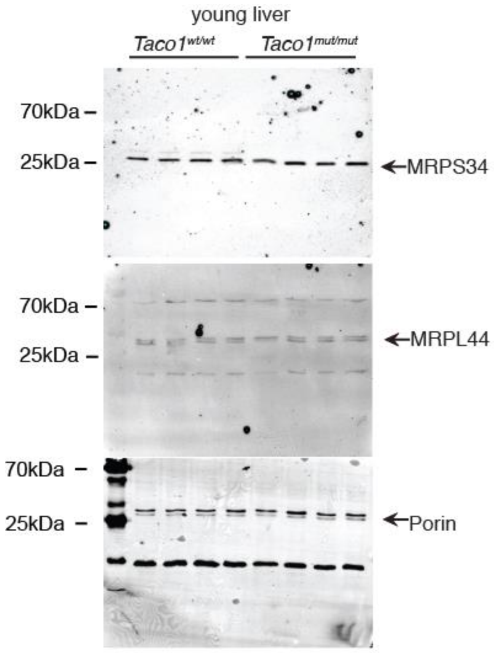

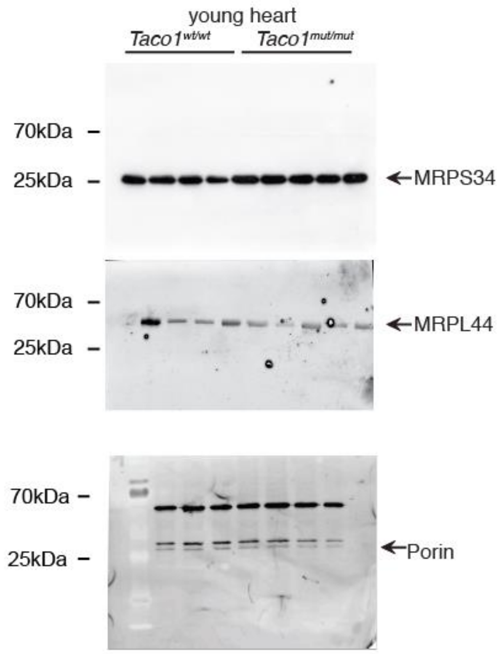

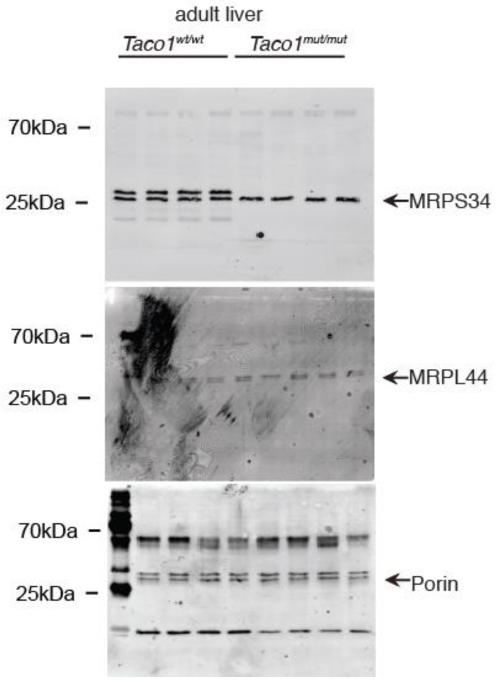

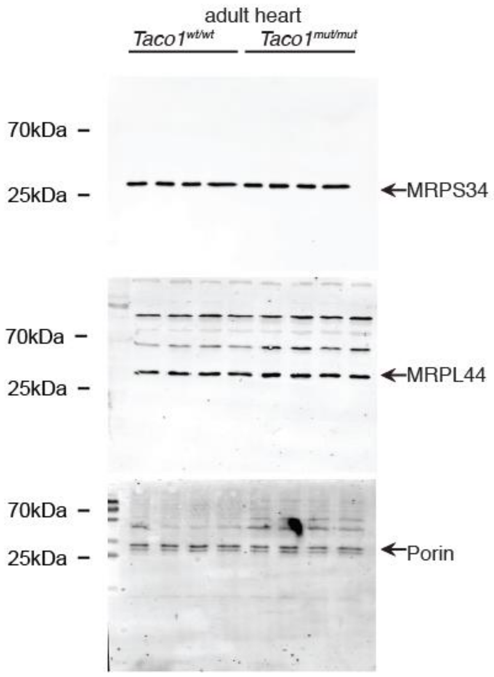

Supplementary Table 1. Data collection and refinement statistics

	Native	Se-Met
**Data collection**		
Space group	$P2_12_12_1$	$P2_12_12_1$
Cell dimensions		
a, *b*, *c* (Å)	48.5, 55.3, 92.0	48.6, 54.7, 91.5
α, β, γ (°)	90, 90, 90	90, 90, 90
Resolution (Å)	100–2.0 (2.07-2.0)*	47.0–2.15 (2.23–2.15)
R_{sym} (%)	5.6 (110.6)	9.7 (157.9)
$I/\sigma(I)$	17.41 (1.73)	15.1 (1.6)
Completeness (%)	99.9 (99.8)	100.0 (100.0)
Redundancy	6.3 (6.4)	12.8 (12.7)
**Refinement**		
Resolution (Å)	19.5–2.0 (2.09-2.00)	
No. reflections	17324	
R_{work} / R_{free} (%)	20.2/22.1	
No. atoms		
Protein	1713	
Water	56	
B-factors		
Protein	57.6	
Water	48.9	
R.m.s. deviations		
Bond lengths (Å)	0.013	
Bond angles (°)	1.062	

Two crystals were used for the structure.
*Values in parentheses are for highest-resolution shell.

Supplementary Table 2. Structure Database Search

PDB-chain	Z	rmsd	lali	nres	% id	Description
4f3q-A	21.5	3.1	211	234	24	YEBC family protein Cbu_1566
1lfp-A	20.0	5.1	211	243	32	Hypothetical protein Aq_1575
1mw7-A	18.6	4.8	210	220	26	Hypothetical protein HP0162
1kon-A	17.9	4.7	198	233	29	Protein YEBC
3hul-A	6.9	6.5	102	277	11	Homoserine kinase
4czd-B	6.9	10.0	84	159	8	Putative transcriptional regulator, ASNC family
4pwu-B	6.9	2.8	67	77	3	Modulator protein MZRA
3wz0-A	6.9	2.4	68	104	12	Ribonuclease P protein component 3
3bic-B	6.9	16.8	70	715	9	Methylmalonyl-COA mutase, mitochondrial precursor
3ofe-B	6.9	68	68	78	9	LDLR Chaperone Boca
4un1-D	6.8	12.7	99	157	8	Putative transcriptional regulator, ASNC family

Results obtained using DaliLite v.3 [7].

Supplemental Methods:

Mass spectrometry based proteomic analysis of affinity purified protein complexes

Purified proteins were subjected to TCA precipitation and resuspended in 10 µl lysis buffer (6 M guanidinium chloride, 10 mM Tris(2-carboxyethyl)phosphine hydrochloride, 40 mM chloroacetamide and 100 mM Tris-HCl). Samples were diluted ten times with 20 mM Tris pH 8.3 and digested overnight at 37°C with 300 ng of Trypsin Gold (Promega). Peptides were desalted using home-made StageTips (Empore Octadecyl C18, 3M; [8]) and eluted with 80 µl of 60% acetonitrile/0.1% formic acid buffer. Samples were dried with a vacuum concentrator plus (Eppendorf) and resuspended in 4 µl of 0.1% formic acid for mass spectrometry using an EASY nano-LC 1000 (Thermo Scientific) coupled via a nano-electrospray ionization source to an Orbitrap Fusion mass spectrometer (Thermo Fisher Scientific). Protein and peptide identification was performed using Peaks [9] version 7.5. Raw data was searched against the mouse reference proteome from UniProt. Label free quantification was performed using Skyline [10] employing the MS1 filtering approach [11]. Statistical analyses were performed in R (R Development Core Team, 2011, available online at http://www.R-project.org/). Only proteins with adjusted p values of less than 0.05 (1% FDR) were termed significant.

Supplemental References:

1. Zschocke, J. HSD10 disease: clinical consequences of mutations in the HSD17B10 gene. *J Inherit Metab Dis* **35,** 81–89 (2012).
2. Lopez Sanchez, M. I. *et al.* RNA processing in human mitochondria. *Cell Cycle* **10,** 2904–2916 (2011).
3. Antonicka, H. & Shoubridge, E. A. Mitochondrial RNA Granules Are Centers for Posttranscriptional RNA Processing and Ribosome Biogenesis. *Cell Rep* **10,** 920–932 (2015).
4. Borowski, L. S. L., Dziembowski, A. A., Hejnowicz, M. S. M., Stepien, P. P. P. & Szczesny, R. J. R. Human mitochondrial RNA decay mediated by PNPase-hSuv3 complex takes place in distinct foci. *Nucleic Acids Res* **41,** 1223–1240 (2012).
5. Castello, A. *et al.* Insights into RNA biology from an atlas of mammalian mRNA-binding proteins. *Cell* **149,** 1393–1406 (2012).
6. Lovell, S. C. *et al.* Structure validation by C alpha geometry: phi,psi and C beta deviation. *Proteins* **50,** 437–450 (2003).
7. Holm, L., Kääriäinen, S., Rosenström, P. & Schenkel, A. Searching protein structure databases with DaliLite v.3. *Bioinformatics* **24,** 2780–2781 (2008).
8. Rappsilber, J., Mann, M. & Ishihama, Y. Protocol for micro-purification, enrichment, pre-fractionation and storage of peptides for proteomics using StageTips. *Nat Protoc* **2,** 1896–1906 (2006).
9. Bin Ma *et al.* PEAKS: powerful software for peptide de novo sequencing by tandem mass spectrometry. *Rapid Commun Mass Spectrom* **17,** 2337–2342 (2002).
10. MacLean, B. *et al.* Skyline: an open source document editor for creating and analyzing targeted proteomics experiments. *Nat Commun* **26,** 966–968 (2010).
11. Schilling, B. *et al.* Platform-independent and label-free quantitation of proteomic data using MS1 extracted ion chromatograms in skyline: application to protein acetylation and phosphorylation. *Mol Cell Proteomics* **11,** 202–214 (2012).
